# Activation-induced cytidine deaminase promotes DNA demethylation and expression of the *Ninjurin-2* gene

**DOI:** 10.1242/bio.062001

**Published:** 2026-06-12

**Authors:** Toshiyuki Okada, Yusuke Toyoda, Shigeaki Saitoh

**Affiliations:** ^1^ Department of Immunology, Kurume University School of Medicine, Asahi-machi 67, Kurume, Fukuoka 830-0011, Japan; ^2^ Department of Cell Biology, Institute of Life Science, Kurume University, Asahi-machi 67, Kurume, Fukuoka 830-0011, Japan

**Keywords:** Activation-induced cytidine deaminase, Colonic epithelial cells, DNA demethylation, Nerve injury-induced protein 2

## Abstract

Activation-induced cytidine deaminase (AID) is expressed in germinal center B cells and contributes to somatic hypermutation and isotype switching of immunoglobulins. AID is also reportedly expressed in colonic epithelial cells (CECs) during inflammation. However, the physiological roles of AID in CECs are largely unknown. Here, we identify 619 genes, the expression of which is induced in a colorectal cancer (CRC) cell line (DLD-1) by overexpression of AID. These genes include those associated with the phosphoinositide-3-kinase-protein kinase B, mammalian target of rapamycin and mitogen-activated protein kinase signaling pathways. We focused on the *NINJ2* gene, the expression of which increased more than fivefold in AID-overexpressing DLD-1 cells and decreased in colitis-induced CECs of AID-knockout mice, compared with those of wild-type mice. We demonstrate that AID binds to the promoter/enhancer region of *NINJ2* and induces DNA demethylation, which is often accompanied by transcriptional upregulation. Additionally, the recovery from induced colitis was significantly delayed in AID-knockout mice. These findings collectively suggest that inflammation-increased AID upregulation in CECs enhances *NINJ2* transcription through DNA demethylation, potentially facilitating wound healing during recovery.

## Teaser

**Summary:** This study reveals that activation-induced cytidine deaminase (AID) may promote colonic epithelial regeneration during inflammation by demethylating the *NINJ2* gene, clarifying an epigenetic role of AID.

## INTRODUCTION

Activation-induced cytidine deaminase (AID) is a DNA/RNA editing enzyme belonging to the apolipoprotein B mRNA-editing enzyme catalytic polypeptide-like complex (APOBEC) family. Stimulation of CH12F3-2 cells, a mouse B-cell lymphoma line, with IL-4, TGF-β, and CD40, induces class switch recombination (CSR) from IgM to IgA. During this process, expression of the *Aicda* gene, which encodes AID, is strongly induced in stimulated CH12F3-2 cells (Muramatsu et al., 1999). Following this report, *AID* mutations were identified as the causative gene in patients with hyper-IgM syndrome type 2 (HIGM2), confirming involvement of this gene. HIGM2 is characterized by mutations in the *AID* gene, leading to impaired CSR and somatic hypermutation (SHM) in B cells. These defects result in decreased serum levels of IgG and IgA and an absence of IgV somatic mutations, as well as lymph node hyperplasia and tonsillar hypertrophy (Revy et al., 2000).

AID was initially characterized as a novel member of the RNA-editing deaminase family (Muramatsu et al., 1999), but it also acts directly on DNA (Petersen-Mahrt et al., 2002). AID protein can be divided into three domains. The N-terminal domain containing nuclear localization signals (NLSs) is essential for induction of DNA cleavage. The central domain is required for cytidine-deaminase activity, and the C-terminal domain, which contains nuclear export signals (NESs), is essential for DNA repair (Arakawa et al., 2002; Maul and Gearhart, 2010; Muramatsu et al., 1999). AID induces mismatch mutations by deaminating deoxycytidine (dC) in single-stranded DNA, converting it to deoxyuracil (dU). This deamination triggers mismatch repair (MMR), leading to C-to-T transition mutations. Additionally, AID-mediated deamination of dCs and subsequent MMR can result in DNA demethylation. Methylated dCs are converted to dTs by deamination and are then replaced with non-methylated dCs during the MMR process (Morgan et al., 2004).

Although AID was thought to be expressed only in germinal center B cells, ectopic AID expression was also observed in the *Helicobacter pylori*-infected gastric mucosal epithelium (Shimizu et al., 2014). Moreover, high expression of AID in non-B cells, such as epithelial cells, has also been observed in bacterial infections and inflammation-related diseases, including hepatitis C-infected liver, bladder urothelial cell carcinoma, fibroblast-like synoviocytes isolated from patients with rheumatoid arthritis, and inflammatory bowel disease (IBD) (Endo et al., 2007, 2008; Igarashi et al., 2010; Li et al., 2019b). Among these ailments, IBD is primarily categorized into Crohn's disease (CD) and ulcerative colitis (UC). The pathogenesis of IBD is complex and multifactorial, affected by immune system dysregulation and environmental influences such as viruses, bacteria, diet, and food additives, as well as genetics (Rivas et al., 2011; Zhang and Li, 2014). AID, which is highly expressed in CECs of IBD patients, is thought to induce SHMs in the tumor suppressor gene, *TP53*, potentially contributing to the malignant transformation of epithelial cells (Endo et al., 2008; Eaden et al., 2001; Nonaka et al., 2016). Thus, AID expression in various epithelial cells is induced by nuclear factor (NF)-κB activation through tumor necrosis factor (TNF)-α stimulation and is further enhanced by the Th2 cytokines IL-4, IL-13, and IL-21 (Araki et al., 2019; Endo et al., 2008; Watashi et al., 2013). Despite these observations, the physiological role of ectopic AID expression in CECs, during bacterial infection and inflammation, remains poorly understood.

In this study, we utilized colorectal cancer (CRC) cell lines stably expressing AID and AID-knockout (AID-KO) mice to investigate whether AID expression in CECs under inflammatory conditions influences gene transcription. Molecular analyses revealed that AID expressed in CRC cells binds directly to the *Ninjurin-2* (*NINJ2*) promoter/enhancer and facilitates DNA demethylation, suggesting a potential mechanism by which AID regulates gene expression during inflammation. Furthermore, using a mouse model of colitis, we found that AID deficiency significantly delayed recovery from weight loss following dextran sulfate sodium (DSS)-induced acute colitis, with persistent diarrhea compared to wild-type (WT) mice. These findings suggest that AID-mediated DNA demethylation may contribute to the regulation of epithelial regeneration during inflammation. By determining the epigenetic function of AID in CECs, our study offers new insights into molecular mechanisms underlying epithelial regeneration and may contribute to the development of therapeutic strategies for IBD.

## RESULTS

### Overproduction of AID alters expression of genes in CRC cell lines

To determine whether AID, which is expressed in CECs under inflammatory conditions, promotes DNA demethylation and regulates gene expression, we first examined *AID* mRNA expression in five CRC cell lines using reverse transcriptase quantitative PCR (RT-qPCR) (Fig. S1A). As reported previously, *AID* mRNA expression was confirmed in LoVo and Caco-2. In contrast, its expression level was below the detection limit in T84 and DLD-1. Although endogenous *AID* expression was undetectable in both cell lines, we selected DLD-1 cells for gain-of-function analyses due to their higher transfection efficiency and proliferation rate. Accordingly, we generated two clones from DLD-1, each of which stably expresses either AID-myc-His (AID-myc-His#1 and AID-myc-His#2) or an empty vector (control#1 and control#2). The expression of AID protein in control and AID-overexpressing (AID-OP) cell lines was confirmed by western blotting (Fig. S1B).

**Fig. 1.**
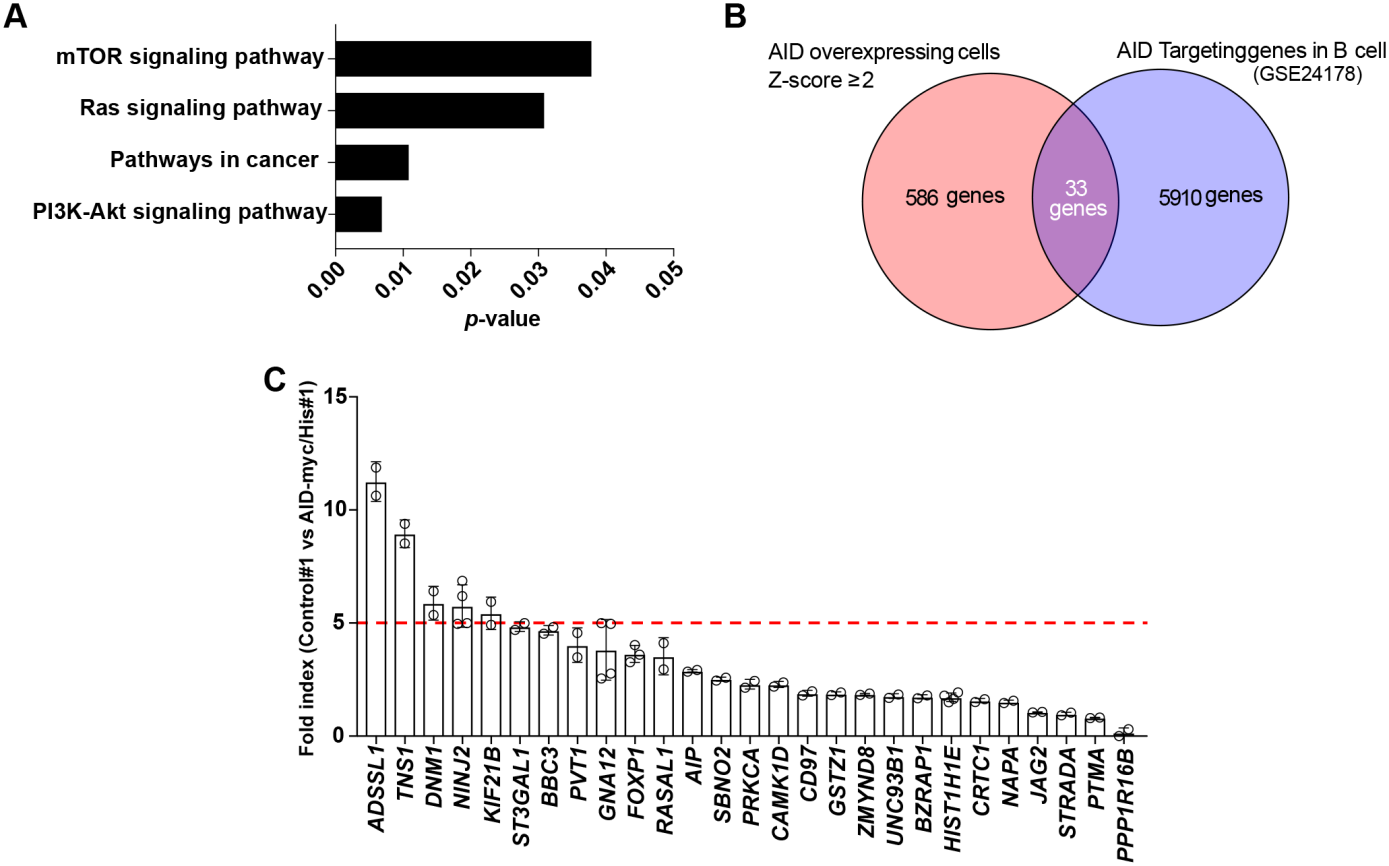
**Identification of genes upregulated in an AID-dependent manner in CRC cells.** For microarray analysis, stable AID- or empty-vector-expressing DLD-1 cells (AID-myc-His#1 and control#2) were generated. This analysis was conducted once without biological replicates, and the data are deposited in GEO with the accession number GSE271083. (A) DAVID signaling pathway analysis was performed using 527 genes with a *Z*-score change of ≥2, which were identified as AID-dependent genes. mTOR, mammalian target of rapamycin; PI3K, phosphoinositide-3-kinase. (B) Potential target genes for AID were compared with target genes for AID in activated B cells (GSE24178). (C) Expression of the 27 genes predicted to be bound by AID was examined by RT-qPCR using control and AID-OP cells. Relative expression of each gene in AID-myc-His#1 cells in comparison to control#1 cells is shown. RT-qPCR was performed using cDNA generated from independent cell cultures. Each measurement was conducted in a single well. The number of biological replicates was as follows: *n*=4 for *NINJ2* and *HIST1H1E*, *n*=3 for *FOXP1*, and *n*=2 for all other genes. Individual data points are plotted as open circles, and bar graphs represent mean±s.d.

Next, the AID-myc-His#1 and control#2 cell lines were used for comprehensive expression analysis by DNA microarray. The candidate genes, the expression levels of which are affected by AID overexpression, were identified using a *Z*-score cutoff (Quackenbush, 2002), and the mRNA levels of the identified candidate genes were subsequently examined by RT-qPCR (see Materials and Methods for details). We generated a heat map of genes with *Z*-scores that increased more than twofold in AID-myc-His#1 cells compared with control#2 cells (Fig. S1C). As described below, AID overexpression in DLD-1 cells induces broad transcriptional changes, leading to the transcriptional upregulation of a defined set of genes, including *NINJ2*. We identified 619 genes with a *Z*-score change of ≥2 in mRNA expression in an AID-dependent manner. The genes are listed in the supplementary material. As DNA demethylation generally increases gene transcription (Moore et al., 2013), we focused on 619 genes, the expression of which increases, to determine the role of AID. Of the 619 genes, 527 had ENTREZ IDs. DAVID functional annotation analysis of these 527 genes revealed that AID expression augmented the expression of genes associated with cancer signaling pathways (*P*=0.011). In addition, phosphoinositide-3-kinase (PI3K)-protein kinase B (Akt, *P*=0.007), mammalian target of rapamycin (mTOR, *P*=0.038), and Ras (*P*=0.031) signaling pathways were substantially enriched (Fig. 1A).

We next assessed whether any of these genes with AID-dependent expression changes are known AID target genes in B cells. In AID-myc-His#1 cells, we identified 33 potential target genes of AID by comparing the 619 activated genes with reported AID target genes in B cells (Yamane et al., 2011) (Fig. 1B). We investigated whether expression levels of these 33 genes increased in AID-OP cells compared with those in control cells by RT-qPCR. Notably, five of the 33 genes, *ADSSL1*, *TNS1*, *DNM1*, *NINJ2*, and *KIF21B*, exhibited ≥5-fold changes in expression level in AID-OP cells (#1 and #2) compared with control cells, in an AID-dependent manner (Fig. S2A). Levels of six genes, *ITM2C*, *NKD2*, *RPL7A*, *TREX1*, *KLF3*, and *PRR7*, could not be quantified by qPCR due to primer selection failure. Data summarizing expression levels of 27 genes in AID-myc-His#1 and AID-myc-His#2 relative to control#1 cells are shown in Fig. 1C and Fig. S2B, respectively. These results suggest that AID expression upregulates the expression of genes related to cell proliferation, survival, and inflammatory responses.

### Loss of AID expression reduces *Ninj2* expression in CECs from AID-KO mice with DSS-induced colitis

**Fig. 2.**
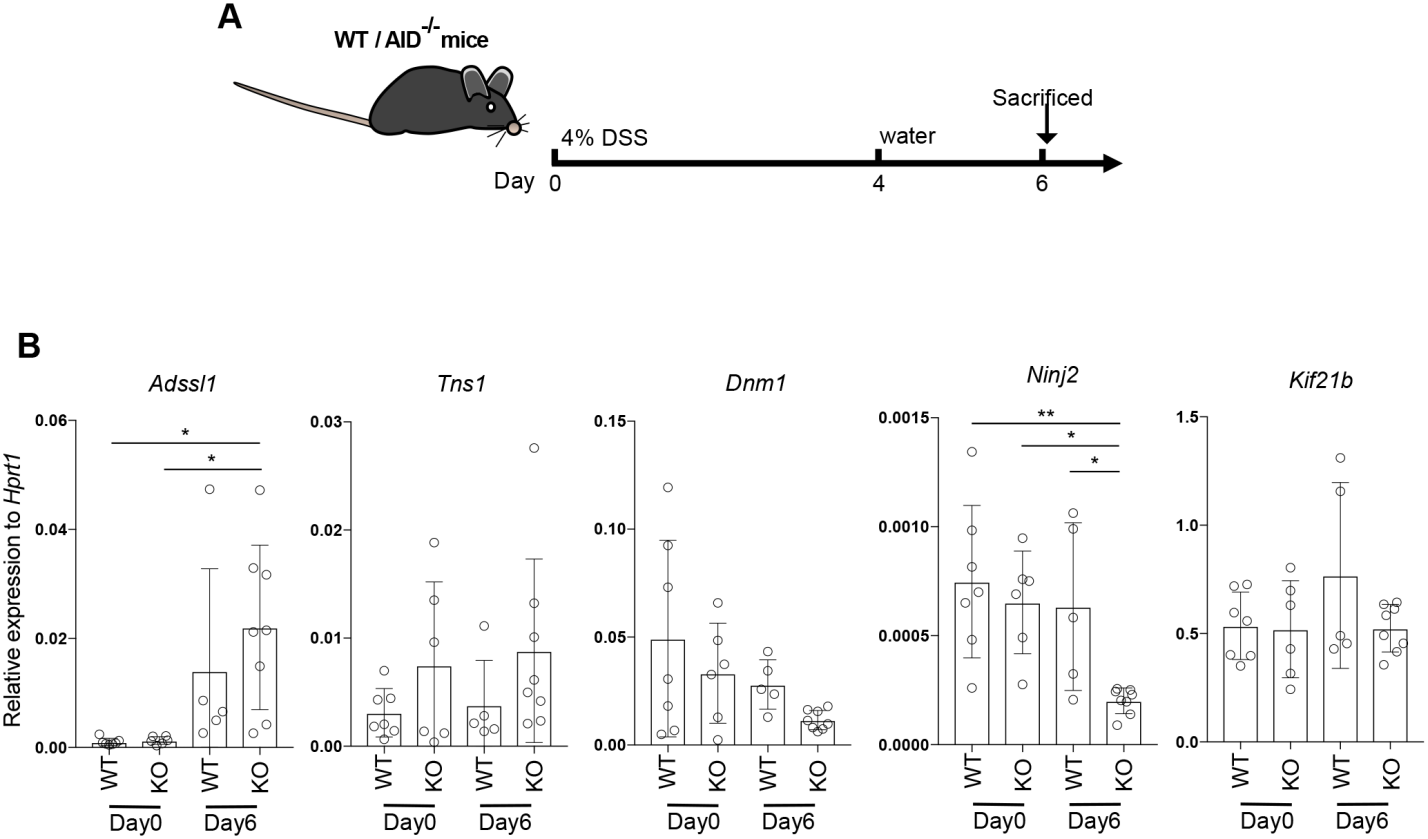
***Ninj2* is upregulated in acute DSS-induced colitis in an AID-dependent manner.** (A) Experimental schedule for the acute DSS-induced colitis mouse model. (B) DSS-untreated mice (day 0) and mice treated with DSS for 6 days (day 6) and then euthanized. Colonic crypts were isolated from WT and AID-KO mice, and mRNA expression levels of five genes (*Adssl1*, *Tns*1, *Dnm1*, *Ninj2*, and *Kif21b*) were confirmed by RT-qPCR, which was performed in technical duplicate. Each open circle represents the result obtained from an individual mouse. The numbers (*n*) of mice analyzed are as follows: *n*=7 for WT at day 0, *n*=6 for KO at day 0, *n*=5 for WT at day 6 and *n*=8 for KO at day 6. Statistical significance was determined using ordinary one-way ANOVA with Tukey's multiple-comparison test (*P<0.05, **P<0.01). The exact *P*-value for *Ninj2* expression in WT versus AID-KO on day 6 is *P*=0.049. Data represent mean±s.d. of results pooled from at least three independent experiments. Individual data points are plotted. WT, wild type; KO, knockout.

As described above, overproduction of AID greatly increases mRNA levels of *ADSSL1*, *TNS1*, *DNM1*, *NINJ2*, and *KIF21B* in CRC cell lines. Since inflammation causes elevated expression of AID in CECs (Endo et al., 2008), these results suggest that the expression of these five genes is upregulated in CECs during intestinal inflammation. To examine this possibility, we used a mouse model in which colitis was induced with DSS and obtained results suggesting that inflammation-induced AID upregulates *Ninj2* expression. First, we administered 4% DSS in drinking water to WT and AID-KO mice *ad libitum* for 4 days to induce acute colitis, followed by normal drinking water for 2 days (Fig. 2A). On day 6, WT and AID-KO mice were euthanized. CECs were purified, and mRNA levels of the five genes were measured by RT-qPCR. CEC expression levels of *Ninj2* mRNA were significantly reduced in AID-KO mice compared with those in WT mice at day 6 (*P*=0.049). On the other hand, *Adssl1* mRNA expression levels were significantly increased in AID-KO mice with acute colitis compared with WT and AID-KO mice in steady-state conditions, as well as WT mice with acute colitis (*P*=0.0005, *P*=0.0010, *P*=0.0168). However, no differences were observed in *Tns1*, *Dnm1*, or *Kif21b* (Fig. 2B). These results suggest that inflammation-induced AID expression is associated with the upregulation of *Ninj2* gene expression *in vivo*.

### Inhibition of AID expression by short interfering RNA (siRNA) attenuates *NINJ2* expression

**Fig. 3.**
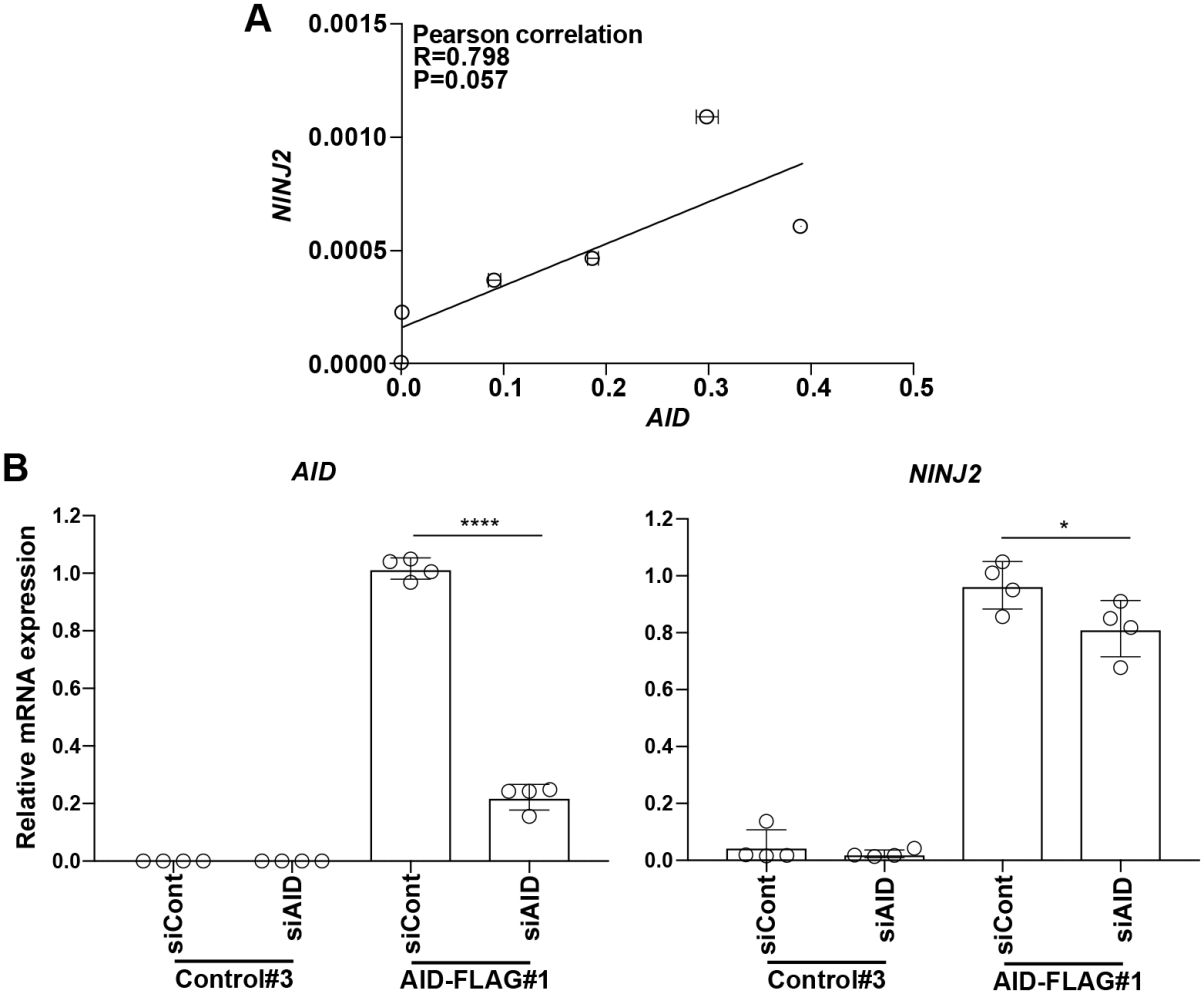
**Knockdown of AID by siRNA attenuates *NINJ2* expression.** (A) Correlation between *NINJ2* and *AID* expression in two control cell clones and four AID-OP cell clones was analyzed using Pearson correlation analysis (*r*=0.798, *P*=0.057). Each sample is indicated by an open circle. Error bars represent the standard error of mean (s.e.m.). (B) control#3 and AID-FLAG#1 cells transfected with siRNA [negative control (siCont) or AID (siAID)] in which expression levels of *AID* and *NINJ2* (relative to negative control in AID-FLAG#1 cells) were verified by RT-qPCR, which was performed in technical triplicates. The mRNA levels were measured in four independent cultures of each cell clone. The open circles represent the results for each culture. Statistical significance was determined using one-way ANOVA with Tukey's multiple-comparison test (*P<0.05, ****P<0.0001). The exact *P*-value for *NINJ2* expression in siCont versus siAID in AID-FLAG#1 cells is 0.047. Data represent mean±s.d. of results pooled from at least three independent experiments. Individual data points are plotted.

As shown above, *in vitro* experiments suggest that AID positively regulates *NINJ2* expression. To further substantiate this finding, we examined the correlation between *AID* and *NINJ2* expression using two control cell clones and four AID-OP cell clones. We found that *NINJ2* expression levels also tended to increase in concert with *AID* expression levels, although this correlation did not reach statistical significance (Pearson correlation coefficient, *r*=0.798, *P=*0.057, Fig. 3A). Therefore, we next investigated the effect of siRNA-mediated knockdown of *AID* on *NINJ2* mRNA expression levels in control#3 and AID-FLAG#1 cells. First, the siRNA of *AID* (siAID) or negative control (siCont) was transfected into control#3 and AID-FLAG#1 cells after 3 days of culture. We then evaluated the expression levels of *AID* in control#3 and AID-FLAG#1 cells by RT-qPCR and found that they were reduced by approximately 80% (*P*<0.0001, Fig. 3B, left). Subsequently, the effect of siRNA knockdown of *AID* on *NINJ2* expression was tested, and *NINJ2* expression levels were reduced by approximately 19% in siAID-transfected AID-FLAG#1 cells compared with siCont-transfected AID-FLAG#1 cells (*P=*0.047, Fig. 3B, right). Together, these findings support a role for AID in positively regulating *NINJ2* expression in CRC cells.

### AID expressed in CRC cells binds to the promoter/enhancer region of *NINJ2*

**Fig. 4.**
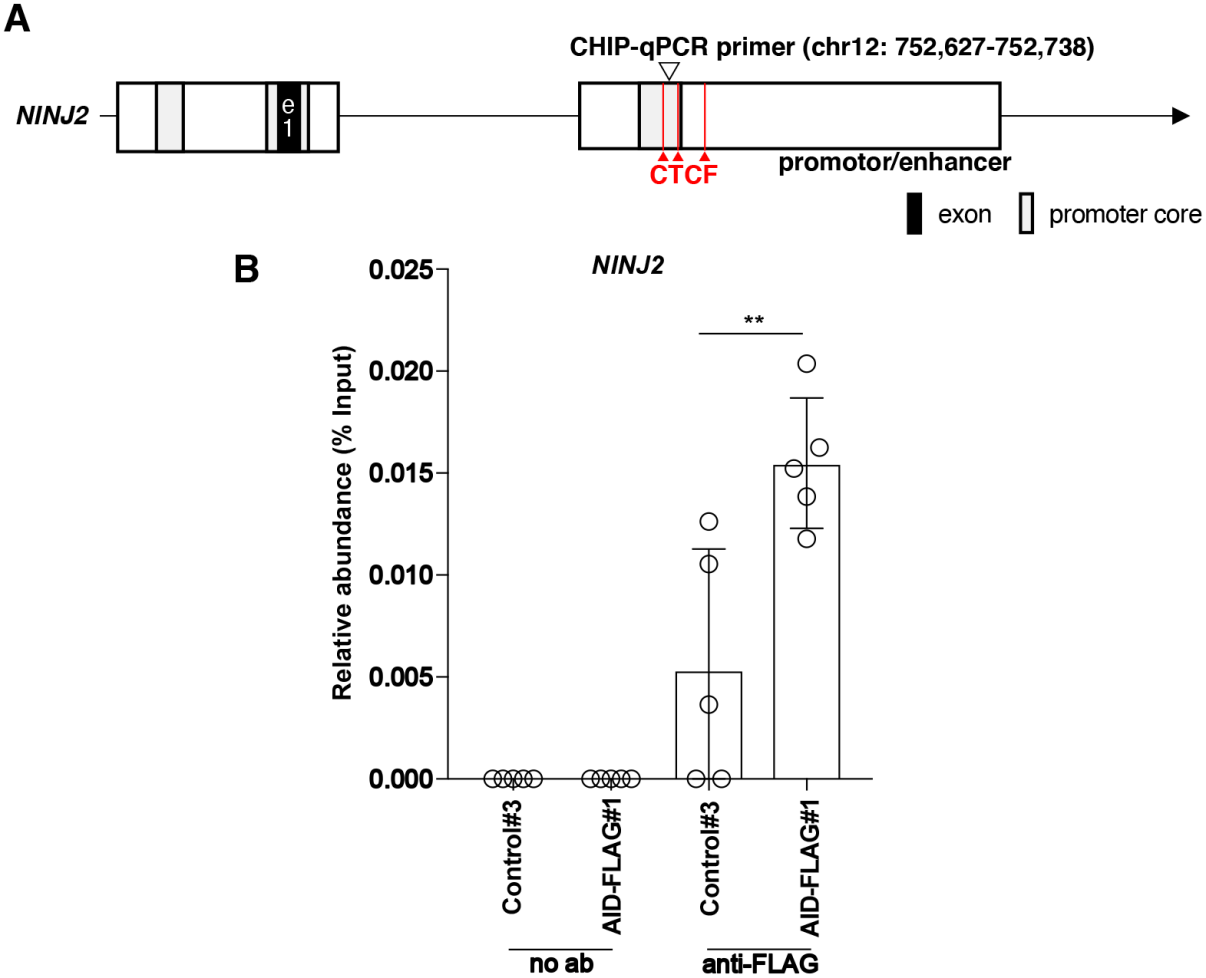
**AID binds to the *NINJ2* promoter/enhancer region.** (A) Genomic map of *NINJ2* and positions of primers used for ChIP-qPCR are shown. (B) ChIP-qPCR analysis of AID binding to the *NINJ2* promoter/enhancer. Chromatin DNA from control#3 (empty-vector-transfected cells) and AID-FLAG#1 cells was immunoprecipitated with anti-FLAG antibody. Mock immunoprecipitation without antibody (no ab) was performed in parallel to confirm that DNA does not bind to magnetic beads nonspecifically. The amount of the *NINJ2* promoter/enhancer region DNA in input and immunoprecipitated DNA was quantified by a single or a duplicated qPCR. The abundance of *NINJ2* DNA in the immunoprecipitated DNA was normalized by dividing it by the abundance of *NINJ2* in the input DNA. The normalized value is presented as % Input. The experiments were repeated five times using independent cell cultures, and each result is represented by an open circle. Bar graphs present mean±s.d. Statistical significance was determined using one-way ANOVA with Tukey's multiple-comparison test (**P<0.01). No ab, no antibody; anti-FLAG, anti-FLAG antibody.

To investigate the mechanism underlying AID-mediated upregulation of NINJ2, we employed chromatin immunoprecipitation (ChIP) to examine whether AID could physically interact with the *NINJ2* promoter/enhancer. As detailed below, our data indicate that AID binds directly to the promoter/enhancer region of the *NINJ2* gene. Since ChIP-grade anti-AID antibodies are not commercially available and the ChIP experiment is highly sensitive and because the anti-Myc antibody may recognize endogenous c-Myc protein in addition to AID-myc-His, we used cell lines stably expressing AID with a 3× FLAG tag at the C-terminus (AID-FLAG#1) or empty vector (control #3) rather than AID-myc-His cell lines. We checked the expression (Fig. S3A) and localization (Fig. S3B) of AID protein and the mRNA expression of five identified genes (*ADSSL1*, *TNS1*, *DNM1*, *NINJ2*, and *KIF21B*) in control#3 and AID-FLAG#1 (Fig. S3C). Hotspot mutations caused by AID accumulate at loci that form G-quadruplex (G4) DNA structures (Xu et al., 2020), and G4 DNA frequently co-localizes with the CCCTC-binding-factor (CTCF) binding site in CpG islands (Hou et al., 2019). Therefore, we designed primers for ChIP-qPCR in the G4-forming region near the CTCF site in *NINJ2* (Fig. 4A). ChIP-qPCR analysis demonstrated significant enrichment of AID at the *NINJ2* promoter/enhancer in AID-FLAG#1 cells compared with control#3 and no-antibody controls [one-way analysis of variance (ANOVA) with Tukey's post hoc test, *P*=0.0011; Fig. 4B]. These results support our hypothesis that AID expressed in CECs under inflammatory conditions binds to the *NINJ2* promoter/enhancer, facilitating its transcriptional regulation.

### AID expression leads to DNA demethylation of the promoter/enhancer region of *NINJ2*

**Fig. 5.**
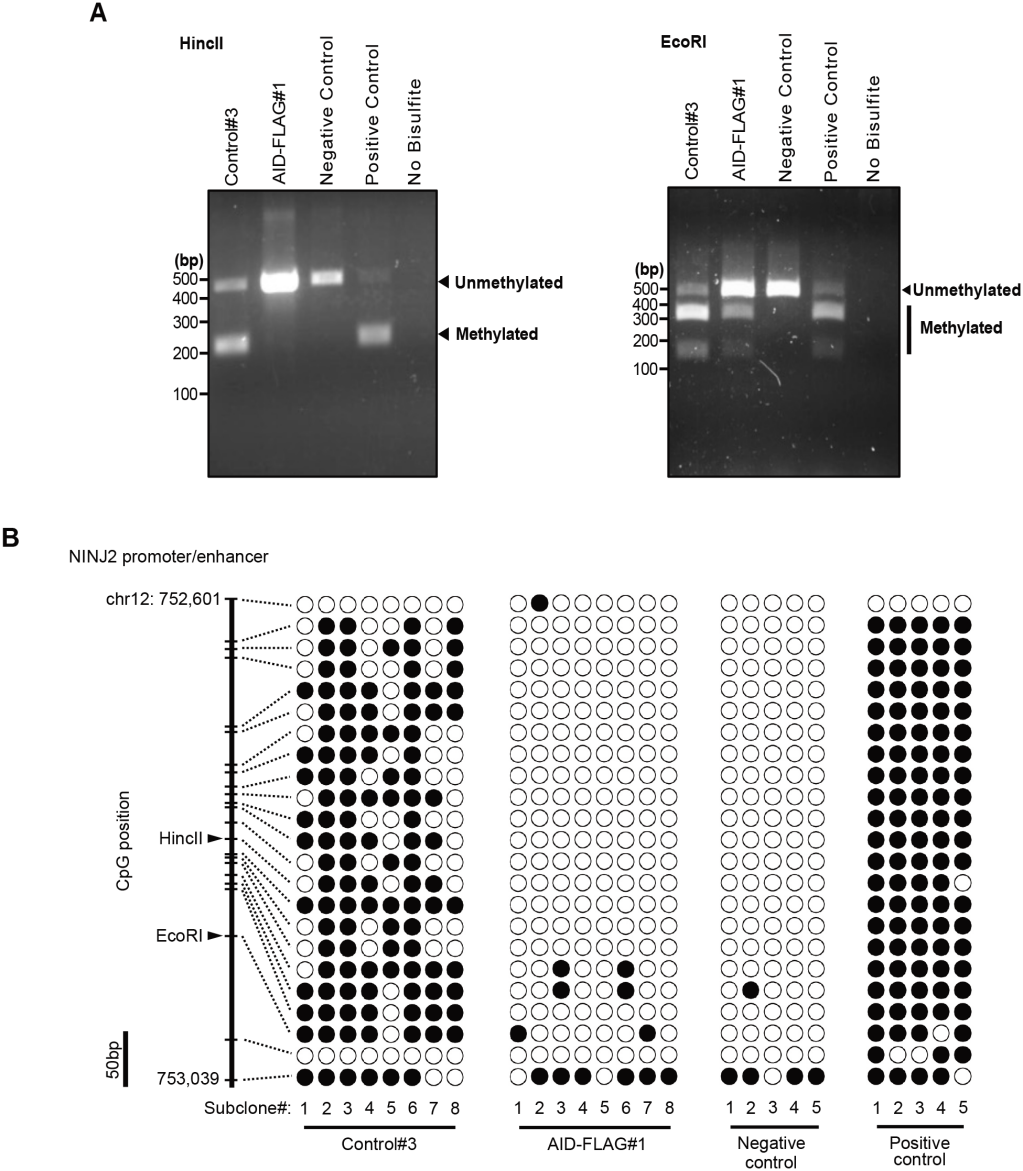
**Overexpression of AID induces DNA demethylation of the *NINJ2* promoter/enhancer region.** (A) COBRA analysis of *NINJ2* methylation in control#3 (empty-vector-transfected cells) and AID-FLAG#1 cell lines. PCR products were digested with *Hin*cII (left) or *Eco*RI (right). These enzymes recognize sequences unique to methylated and bisulfite-unconverted alleles. To validate assay specificity, unmethylated DNA (negative control) and fully methylated DNA (positive control) from the EpiTect PCR control kit (QIAGEN) were used. No bisulfite (non-bisulfite-treated DNA) was also used as a control for bisulfite treatment. Data are representative of three independent experiments. (B) Methylation status of *NINJ2* in control#3 and AID-FLAG#1 cells according to bisulfite sequencing. Locations of 23 CpG positions in the sequenced region (chr12: 752,601-753,039) in the *NINJ2* gene, ChIP-qPCR primers, and *Hin*cII and *Eco*RI cleavage sites used in COBRA are shown. BSP data analysis was performed using the web-based tool QUMA (Quantification Tool for Methylation Analysis), and the methylation status of each CpG in the region is shown as open circles (non-methylated DNA) or closed circles (methylated DNA). The gDNA was isolated from single cultures of the control#3 and AID-FLAG#1 cell lines. Eight subcloned DNA fragments from the PCR amplicon of each experimental group were sequenced, whereas five subcloned DNA fragments were sequenced for the controls. BSP, bisulfite sequencing PCR.

We hypothesized that AID-mediated deamination induces DNA demethylation, leading to enhanced gene expression. To investigate how AID regulates *NINJ2* expression, we utilized combined bisulfite restriction analysis (COBRA) and bisulfite sequencing PCR (BSP) to examine and verify the methylation status of the *NINJ2* promoter/enhancer. Our results suggest that AID expression promotes DNA demethylation of the *NINJ2* promoter/enhancer, as described below. First, we extracted genomic DNA (gDNA) from control#3 and AID-FLAG#1 cells. This DNA was used to convert bisulfite-modified DNA to reveal the methylation status of regions (chr12: 752,627-752,738) amplified by ChIP-qPCR. Bisulfite-modified DNA was amplified by PCR as a template, and PCR products were subjected to restriction enzyme (*Hin*cII or *Eco*RI) treatment at 37°C for 5 h to determine methylation status. Bisulfite treatment converts unmethylated C to U, which is subsequently converted to T after PCR amplification. Thus, the 6 bp sequence recognized by *Hin*cII or *Eco*RI, which contains C (GTYRAC, where Y=T or C and R=A or G, or GAATTC), becomes unrecognizable by *Hin*cII or *Eco*RI if it is not methylated. Treatment with *Hin*cII (Fig. 5A, left) and *Eco*RI (Fig. 5A, right) revealed that the promoter/enhancer region of *NINJ2* was hypermethylated in control#3 cells but hypomethylated in AID-FLAG#1 cells. Therefore, to confirm the results of COBRA, we verified the methylation status of *NINJ2* by BSP analysis. Consistent with the results of COBRA, BSP analysis also revealed markedly reduced methylation of DNA in the promoter/enhancer region (chr12: 752,601-753,039) of *NINJ2* in AID-FLAG#1 cells compared with control#3 cells (Fig. 5B). These results indicate that AID promotes DNA demethylation of the *NINJ2* promoter/enhancer, thereby enhancing its transcription.

### AID deficiency delays recovery from colitis and exacerbates inflammation

**Fig. 6.**
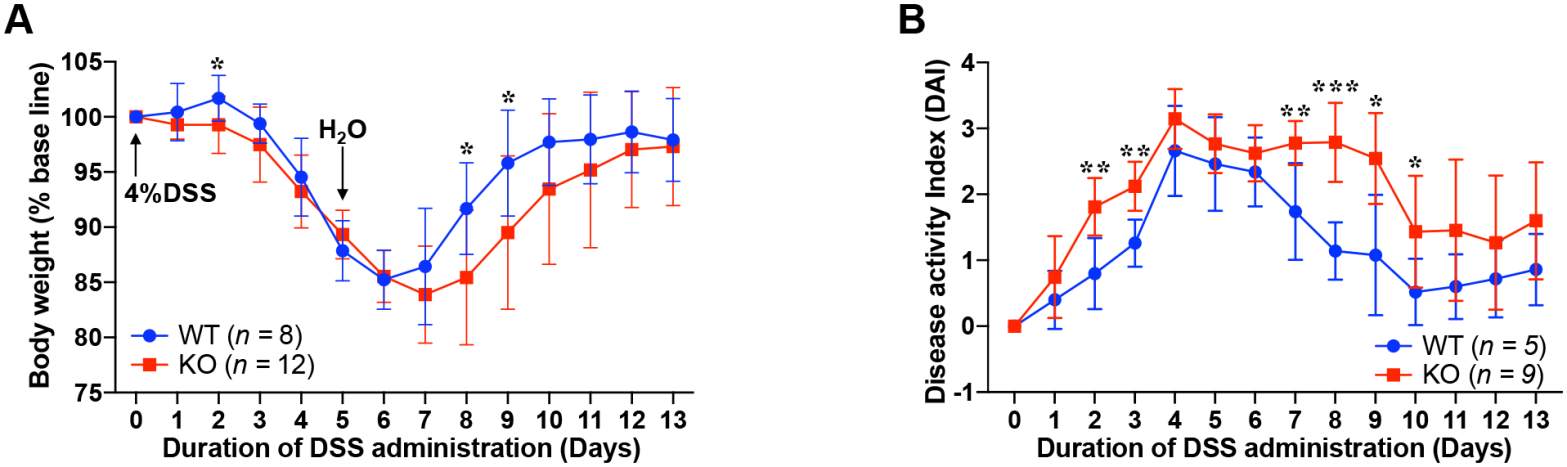
**Loss of colitis-induced AID exacerbates the severity of colitis.** (A) Changes in body weight of WT (*n*=8) and AID-KO (*n*=12) mice treated with 4% DSS for 4 days, followed by normal drinking water until day 13. To minimize inter-individual variability, mice exhibiting a 10-20% reduction in body weight on day 6 were selected for analysis. (B) In a subset of these mice, DAI scores were monitored daily (WT, *n*=5; AID-KO, *n*=9). The DAI was calculated by scoring body weight, blood stools, and diarrhea according to Table S6 and dividing by three to derive the mean value. *n* indicates the number of mice used for each genetic background. Blue and red lines indicate WT and AID-KO mice, respectively. Statistical significance was determined using the nonparametric Mann-Whitney *U* test. KO, knockout; WT, wild type (*P<0.05, **P<0.01, ***P<0.001). The exact *P*-value for the DAI score on day 10 is 0.049. Data represent mean±s.d. of results pooled from at least three independent experiments.

Considering the role of AID in transcriptional regulation, we further investigated its physiological significance, particularly in epithelial regeneration. Since goblet cell recovery and colonic epithelial regeneration occur in a DSS-induced colonic mucosal injury model (Sugimoto et al., 2008), we utilized this model to assess the effect of AID loss on epithelial regeneration during recovery from DSS-induced intestinal injury. As detailed below, AID deficiency significantly delays mucosal healing. In the 4% DSS recovery model, mice exhibiting comparable initial injury (10-20% body weight loss on day 6) were selected to minimize variability. Body weight, stool consistency, and existing gross blood in feces were monitored daily and scored (Table S6) until day 13 (Fig. 6A). We calculated the disease activity index (DAI) score using the method described by Cooper et al. (1993) and Hidalgo-Cantabrana et al. (2016). During the recovery phase (days 8 and 9), AID-KO mice exhibited significantly delayed body weight recovery compared with WT mice (*P*=0.024, *P*=0.041, Fig. 6A). Furthermore, diarrhea persisted significantly longer in AID-KO mice on days 7, 8, and 9 but was not observed in WT mice (*P*=0.005, *P*=0.0015, *P*=0.015, Fig. S4A). However, no significant differences were observed in rectal bleeding (Fig. S4B). These results suggest that inflammation is more severe in AID-KO mice on days 7, 8, 9, and 10, as indicated by significantly higher DAI scores compared with WT mice (*P*=0.006, *P*=0.0005, *P*=0.013, *P*=0.049, Fig. 6B).

To further validate these findings in a model with intrinsically lower variability, we also performed experiments using a mild dose (2%) of DSS. Consistent with the 4% DSS model, AID-KO mice in the 2% DSS group showed greater body weight loss (days 6-9) and delayed recovery compared with WT mice (*P*=0.02, *P*=0.02, *P*=0.02, *P*=0.039, Fig. S4C). DAI scores on days 5, 9, and 10 were also significantly higher in AID-KO mice (*P*=0.048, *P*=0.035, *P*=0.004, Fig. S4D). Taken together, these results suggest that inflammation-induced AID expression in CECs contributes to epithelial regeneration and recovery from intestinal injury.

## DISCUSSION

**Fig. 7.**
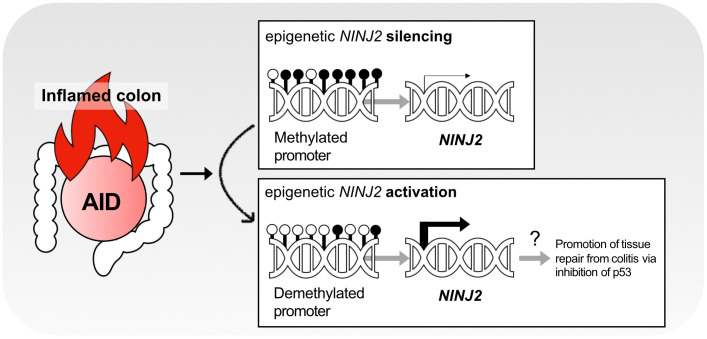
**Schematic representation of AID-driven *NINJ2* transcription during inflammation.** Normally, AID is not expressed in CECs; however, its expression is induced during inflammation. We demonstrate that inflammation-induced AID binds to the promoter/enhancer region of *NINJ2* and triggers DNA demethylation. Based on these findings, we propose a model in which AID-mediated transcriptional regulation of *NINJ2* plays a physiological role in promoting recovery from colitis.

In this study, we show that AID expressed in CECs under inflammatory conditions upregulates *NINJ2* expression by binding to promoter/enhancer regions of *NINJ2*, reducing its DNA methylation levels. Based on these findings, we propose a model in which AID expression, induced by intestinal injury, may be involved in the recovery process from acute intestinal inflammation (Fig. 7).

While AID expressed during chronic inflammation is thought to act as a mutator that drives carcinogenesis (Endo et al., 2008; Takai et al., 2012), the results shown here reveal its role in transcriptional regulation by DNA demethylation. DNA methylation is a crucial epigenetic modification that regulates gene expression, primarily by suppressing gene activity through methylation of promoter regions. This modification prevents binding of transcription factors and RNA polymerase. Conversely, DNA demethylation enhances gene expression by increasing chromatin accessibility and by facilitating binding of regulatory elements (Hon et al., 2014; Kribelbauer et al., 2020; Pandiyan et al., 2013). The APOBEC family, including AID, contributes to DNA demethylation through deamination of 5mC to thymine (Thy) and 5hmC to 5hmU (Morgan et al., 2004; Ramiro and Barreto, 2015). These deaminated products are processed by DNA repair mechanisms, promoting DNA demethylation (Barreto et al., 2007; Cortellino et al., 2011; Hendrich et al., 1999; Jiao et al., 2023; Rai et al., 2008). Thus, AID-induced deamination is expected to trigger both somatic mutations and DNA demethylation. Since AID induces SHM in immunoglobulin variable regions and hotspot mutations in non-immunoglobulin genes such as *TP53* (Takai et al., 2012), its demethylating activity is likely confined to specific cytosine residues, whereas broader demethylation patterns may occur through the action of other demethylating enzymes and co-factors. Recently, Fazio et al. (2022) showed that DNMT3A dysfunction, which is important for DNA methylation and its maintenance, is critical for intestinal homeostasis and intestinal barrier function. In addition, AID expressed in prostate cancer induces CXCL12 demethylation and contributes to cancer metastasis (Li et al., 2023). These findings support the possibility that altered DNA methylation modulated by AID contributes to tissue regeneration from colitis. In summary, AID, the expression of which is induced by inflammation, is involved in both somatic mutation and DNA demethylation through cytosine deamination and may serve an adaptive function in the maintenance of homeostasis.

Furthermore, Kakiuchi et al. (2020) reported that introducing mutations in genes of the IL-17 signaling pathway, which is involved in inflammation to maintain intestinal homeostasis in patients with IBD, resulted in survival, proliferation, and positive selection of crypts resistant to inflammation. Interestingly, the mutation is enriched in cytosine residues. In the future, clarifying how AID expression under inflammatory conditions regulates somatic mutation and DNA demethylation may help to explain mechanisms of epithelial regeneration and chronic inflammatory carcinogenesis.

We found that AID is required for transcriptional upregulation of *Ninj2* upon colitis induction by DSS and that loss of AID significantly delays recovery from colitis in mice. These findings suggest that *NINJ2* may be associated with tissue regeneration during recovery from intestinal injury. However, the causal relationship between *NINJ2* expression and recovery has yet to be demonstrated. *NINJ2*, identified in this study as an AID-mediated demethylation target, was initially characterized as an injury-induced molecule in Schwann cells (Araki and Milbrandt, 2000). NINJ2 interacts with various signaling molecules, including TLR4 (Wang et al., 2017), TNFR1 (Sun et al., 2022a), ITGB1 (Sun et al., 2022b), IGF1R (Peng et al., 2023; Wang et al., 2023), and TP53 (Zhang et al., 2024). Interestingly, NINJ2 competitively inhibits TNF-α/TNFR1 signaling in oligodendrocytes, thereby reducing MLKL phosphorylation levels, whereas NINJ2 abrogation enhances TNF-α-induced necroptosis (Sun et al., 2022a). Anti-TNF-α therapy, the main therapy for IBD patients, prevents the death of CECs and contributes to mucosal healing and regeneration (Neurath, 2014). Zhou et al. (2021) also showed that excessive necroptosis contributes to intestinal barrier dysfunction in patients with IBD and in a DSS-induced colitis model. Thus, since NINJ2 can inhibit TNF-α/TNFR1-mediated necroptosis, it is possible that AID may influence necroptosis-related pathways during intestinal inflammation, which could be associated with tissue recovery.

Next, we found that overexpression of AID in the CRC cell line upregulated expression of genes associated with the Ras/MAPK and PI3K-Akt-mTOR pathways, which are crucial for epithelial homeostasis (Fig. 1A). In hepatocytes, NINJ2 interacts with IGF1R to activate the PI3K-Akt-EGR1 pathway, which regulates cell proliferation, differentiation, and survival (Wang et al., 2023). In addition, *NINJ2* knockdown in CRC cells attenuates ERK and AKT phosphorylation, major factors in the EGFR signaling pathway (Li et al., 2019a). Thus, it is also possible that AID-mediated upregulation of *NINJ2* expression may be associated with activation of signaling pathways related to proliferation and survival of CECs; however, whether these changes contribute to epithelial regeneration remains unclear. *NINJ2* expression is induced by DNA damage in a p53-dependent manner and subsequently suppresses both WT and mutant TP53 expression (Zhang et al., 2024). While AID targets p53 for mutagenesis in colitis-associated CRC (CAC) (Endo et al., 2008; Kakiuchi et al., 2020; Nanki et al., 2020; Takai et al., 2012), our findings suggest an alternative possibility: inflammation-induced AID expression may be linked to changes in cell proliferation in CECs, potentially in association with *NINJ2* expression. This proliferative response may contribute to tissue repair but also predispose cells to carcinogenesis. Moreover, given that mutations in the C/EBP beta binding site in the *NINJ2* promoter region in humans, which is associated with inflammatory responses, lead to reduced *NINJ2* mRNA expression (Wang et al., 2021), it is possible that other factors independent of AID also contribute to regulation of *NINJ2* under inflammatory conditions. While we demonstrated epigenetic regulation of *NINJ2* by AID, the causal contribution of *NINJ2* to epithelial regeneration remains to be fully elucidated. To definitively validate this mechanism, rescue experiments, such as re-expressing *NINJ2* in an AID-deficient background, would be valuable. Additionally, *NINJ2* expression may also be influenced by inflammatory signaling pathways, including NF-κB activation, independently of AID.

Considering that AID can induce genomic instability (Ramiro et al., 2006), excessive AID expression in epithelial cells may exert oncogenic effects. Notably, aberrant AID expression in patients with IBD has been implicated in driving somatic mutations in the *TP53* tumor suppressor gene, thereby facilitating malignant transformation (Endo et al., 2008; Eaden et al., 2001; Nonaka et al., 2016). Therefore, distinguishing between regenerative and tumor-promoting processes is essential for understanding the balance between tissue repair and carcinogenesis. Accordingly, further investigation into the precise interplay between inflammation-induced AID and *NINJ2* is warranted to define their roles in colonic epithelial homeostasis.

A limitation of this study is that systemic AID-deficient mice exhibit intestinal dysbiosis due to the absence or low number of B cells producing IgG, IgE and IgA, which may affect epithelial regeneration. AID-deficient mice lack effective immunoglobulin isotype switching, leading to reduced IgA production, microbiota dysbiosis, and systemic immune activation (Fagarasan et al., 2002). Similarly, the gut microbiota of patients with selective IgA deficiency is also reduced below the detection limit for *Eubacterium biforme*, the main butyrate-producing bacterium, and *Prevotella copri*, which contributes to bile acid metabolism (Catanzaro et al., 2019). This suggests that the decrease in butyrate, a major energy source for CECs, may lead to reduced function of the intestinal barrier and reduced numbers of regulatory T cells (Furusawa et al., 2013), which may affect recovery from colitis. Furthermore, bile acid signaling via G-protein-coupled bile acid receptor 1 (*GPBAR1*, also called *TGR5*) promotes intestinal stem cell activation and colonic epithelial regeneration (Sorrentino et al., 2020), which may also affect delayed epithelial regeneration from intestinal injury. Although our *in vitro* experiments clearly demonstrate that AID binds to the *NINJ2* promoter and induces DNA demethylation, it is important to consider that the delayed regeneration observed in AID-deficient mice may also be influenced by systemic immune alterations such as reduced IgA production and dysbiosis. A further limitation of this study is that, although our *in vitro* data elucidate the AID-dependent epigenetic regulation of *NINJ2*, we did not directly demonstrate that *NINJ2* mediates the recovery process *in vivo*. Thus, while a mechanistic link is highly plausible, the definitive causal contribution of *NINJ2* to physiological tissue regeneration remains to be established. To address these concerns, further studies using CEC-specific, AID-conditional KO models, along with *in vivo* rescue experiments such as *NINJ2* re-expression, may be needed to clarify the precise contribution of inflammation-induced AID and *NINJ2* on epithelial regeneration.

## MATERIALS AND METHODS

### Cell cultures

The human CRC cell line DLD-1 (TKG 0379) was provided by the Cell Resource Center for Biomedical Research, Institute of Development, Aging, and Cancer, Tohoku University. Caco-2 and LoVo were provided by the RIKEN BRC Cell Bank (Japan). T84 was purchased from the American Type Culture Collection (ATCC, Manassas, VA). DLD-1 cells were cultured in RPMI 1640 medium (cat#30264-85, Nacalai Tesque) supplemented with 10% heat-inactivated FBS (cat#F7524, Sigma) and 10 mM HEPES (cat#17557-94, Nacalai Tesque). Caco-2 cells were cultured in Eagle's minimum essential medium (MEM; Nacalai Tesque, Kyoto, Japan) supplemented with 20% heat-inactivated FBS (Sigma), 0.1 mM MEM nonessential amino acid (NEAA) solution (Nacalai Tesque), and 1 mM sodium pyruvate solution (Nacalai Tesque). LoVo cells were cultured in Ham's F-12 medium (Nacalai Tesque) supplemented with 10% heat-inactivated FBS (Sigma) and 25 mM HEPES (Nacalai Tesque). T84 cells were cultured in DMEM/F-12 medium (Nacalai Tesque) supplemented with 5% heat-inactivated FBS (Sigma).

### Plasmid construction and transfection

We generated cloning primers (Table S1) based on the human *AID* coding sequence in the Ensembl Genome Browser (GRCh37) and used them for plasmid insertion. PCR amplification was performed using TaKaRa Ex Taq (cat#RR001B, TaKaRa) with 100 ng of cDNA from HeLa cells, 0.2 µM forward and reverse primers, 1×Ex Taq buffer, 0.2 mM of each dNTP, and 1 U of Ex Taq polymerase in water, for a total of 40 µl. PCR conditions were as follows: incubation at 94°C for 2 min, followed by five cycles of 94°C for 30 s, 48°C for 30 s, 72°C for 1 min, followed by 32 cycles of 94°C for 30 s, 55°C for 30 s, 72°C for 1 min, and a final extension reaction at 72°C for 10 min. The PCR product was digested with restriction enzymes (Table S1) for 2 h or overnight at 37°C to insert it into the plasmid. Digested PCR products were electrophoresed in 2% agarose gels in TBE buffer at 135 V for 25 min, and target bands were excised from the gel and purified according to the manufacturer's protocol for the Wizard SV Gel and PCR Clean-Up System (A9285, Promega, Madison, WI, USA). As inserts, DNA fragments and vectors [pcDNA3.1plus (cat#V790-20, Invitrogen), pcDNA3.1plus/myc-His (cat#V80020, Invitrogen), or pEGFP-N1 (cat#6085-1, Clontech)] were mixed at a ratio of 2:1 and then ligated at 16°C overnight using a DNA Ligation kit (Mighty Mix, cat#6023, TaKaRa). After transformation using ECOS™ Competent *Escherichia coli* DH5α (cat#310-06233, NIPPON GENE) according to the manufacturer's protocol, plasmid DNA was extracted from a single colony, and insertion of the human *AID* sequence into the plasmid was verified by direct sequencing. Cloning and sequencing primers used in these experiments are listed in Table S2. In this study, the following plasmids were used as negative controls, depending on the experimental context: control#1, pEGFP-N1; control#2, pcDNA3.1/myc-His; and control#3, pcDNA3.1.

Empty vectors, or those containing AID (untagged) or AID with 3FLAG, were transfected into DLD-1 cells using Lipofectamine 3000 (cat#13778-075, Invitrogen) to generate cells that stably expressed them. Forty-eight hours after transfection, cells were cultured with G418 (800 µg/ml, Nacalai Tesque). At that time, G418 was also added to non-transfected cells to confirm complete selection, and transfected cells were distributed in 96-well plates at one cell per well to obtain single-clone cells. Expression levels of AID in transfectants were confirmed by western blotting (Figs S1B and S3A). To maintain DLD-1 cells stably expressing AID or empty vector, G418 (400 µg/ml, Nacalai Tesque) was added to the culture medium.

In culture medium, 5×10^4^ cells were resuspended, spread onto 24-well plates, and reverse-transfected with 10 nM siAID (cat#021409-02, Dharmacon, Inc.). The target sequence was 5′-GGACUUUGAUAGCAACUUC-3′. As controls, we used KIF11 (Eg5, cat#150427, Ambion) as the positive control and scramble siRNA (cat#150428, Ambion) as the negative control. Transfection was performed using the Lipofectamine RNAiMAX reagent (cat#13778-075, Invitrogen) according to the manufacturer's protocol. After 72 h, cells were collected and purified using an RNeasy Mini kit (cat#74104, QIAGEN).

### DNA microarray analysis

Total RNA for DNA microarray analysis was extracted from DLD-1 cells stably expressing empty vector (control#2) or AID (AID-myc-His#1) using a TRIzol reagent (cat#15596026, Invitrogen) and purified using an SV Total RNA Isolation System (cat#Z3100, Promega, Madison, WI, USA). For a quality check of total RNA used for microarray analysis, the RNA Integrity Number (RIN) was evaluated using an Agilent 2100 bioanalyzer (Agilent, Santa Clara, CA, USA). RIN values of AID-OP and control samples were 7.4 and 8.0, respectively, above the threshold for ideal microarray analysis. cRNA was amplified and labeled using an Agilent Low-Input QuickAmp Labeling Kit, one-color (cat#5190-2305, Agilent, Santa Clara, CA, USA), and was hybridized to a SurePrint G3 Human Gene Expression Microarray 8x60K ver.2.0 following the manufacturer's instructions. Scanning was performed using an Agilent scanner (Agilent, Santa Clara, CA, USA). Relative hybridization intensities and background hybridization values were calculated using the Agilent Feature Extraction Software (9.5.1.1).

Because microarray analysis was performed on a single biological replicate per group, global multiple testing corrections (e.g. FDR) were not applicable. Instead, we employed an intensity-dependent *Z*-score transformation method to identify significantly altered genes (Quackenbush, 2002). First, raw signal intensities were normalized, and the log-ratio (*R*) and log-mean intensity (*I*) were calculated for each gene. To determine whether the expression ratio deviated from the local average, genes were ordered by their intensity (*I*), and a local mean (*µ*) and standard deviation (*σ*) were calculated within a sliding window of neighboring genes. The *Z*-score for each gene was then calculated using the formula *Z*=(*R*−*µ*)/*σ*. Genes with *Z*-scores >2.0 or <−2.0 and fold changes ≥1.5 or ≤0.67 were considered significantly altered. This approach effectively minimizes false positives in low-expression areas by normalizing for intensity-dependent variability. Using these criteria, we identified genes in AID-myc-His#1 cells relative to control#2 cells as AID-specific altered genes.

### qPCR

Purification of total RNA was performed using the same procedure as for DNA microarray analysis, followed by reverse transcription of total RNA into single-stranded cDNA using the SuperScript IV VILO Master Mix with ezDNase (cat#11766050, Thermo Fisher Scientific Inc.). RT-qPCR was performed using cDNA with the FastStart Essential DNA Green Master Mix (cat#06402712001, Roche Diagnostics) and a LightCycler® 96 system (cat#05815916001, Roche Diagnostics). Primer sequences used for RT-qPCR for human and mouse genes are listed in Table S3. All reactions were performed in duplicate or triplicate with a reaction volume of 20 µl at an annealing temperature of 60°C.

### ChIP

After seeding 5×10^6^ cells in 15 cm dishes for 3-4 days, 20 ml of culture medium containing 0.5% formaldehyde, warmed to 37°C, was added, and cells were fixed for 5 min. To stop the reaction of formaldehyde, 0.22 µm filtered glycine (125 mM, Nacalai Tesque) was added, and cells were washed with ice-cold PBS containing 125 mM glycine. Crosslinked cells were collected using a cell scraper, resuspended in ice-cold PBS containing protease inhibitor cocktail (PIC) and 2 mM PMSF, and pelleted at 700×***g*** for 4 min.

After cells were lysed by adding ChIP buffer I [50 mM HEPES/KOH (pH 7.5), 140 mM NaCl, 1 mM EDTA (pH 7.5), 1% Triton X-100, 0.1% sodium deoxycholate] containing PIC and 2 mM PMSF to the pellet, the chromatin DNA was fragmented to ∼150 bp using a Bioruptor (ON: 30 s, OFF: 30 s, high setting, 30 cycles, 4°C; UCD-300, Cosmo Bio). To remove insoluble materials, the cell lysate containing the fragmented DNA was centrifuged at 17,000×***g*** for 15 min at 4°C. The protein concentration of the supernatant was determined using a Bradford Protein Assay Kit I (cat#5000001, Bio-Rad). The supernatant containing 2 mg of protein was precleared by incubating it at 4°C for 1 h with 10 µl of SureBeads Protein A Magnetic Beads (cat#1614013, Bio-Rad). 10% of the precleared supernatant was saved for subsequent purification of total DNA. 1 μg of anti-FLAG antibody (cat#014-22384, clone#1E6, Fujifilm) was added to the precleared supernatant, and the mixture was incubated at 4°C for 1 h. The mixture was then incubated at 4°C for an additional 2 h after the addition of 10 µl of protein A magnetic beads. After pelleting the beads with a magnet, the supernatant was removed, and the beads were washed three times with wash buffer I [50 mM HEPES/KOH (pH 7.5), 500 mM NaCl, 1 mM EDTA (pH 7.5), 1% Triton X-100, 0.1% sodium deoxycholate]. The beads were then washed twice with wash buffer II [10 mM Tris-HCl (pH 8.0), 0.25 M LiCl, 0.5% NP-40, 0.5% sodium deoxycholate] and TE [Tris-EDTA (pH 8.0)]. To remove RNA from chromatin immunoprecipitated DNA, the beads were incubated in TER [Tris-EDTA (pH 8.0), 10 µg/ml RNase A] at 37°C for 1 h.

After the addition of 0.25% sodium dodecyl sulfate (SDS; Nacalai Tesque) and 250 µg/ml of proteinase K (cat#15679-64, Nacalai Tesque), the beads were incubated at 37°C for 8 h to remove proteins and at 65°C for 6 h for de-crosslinking. To purify the total DNA in the cell lysate (input DNA), the saved supernatant was treated similarly to remove RNA and proteins and then incubated at 65°C for 6 h. The ChIP-enriched and input DNAs were extracted with phenol/chloroform/isoamyl alcohol (PCI, 25:24:1, cat#311-90151, NIPPON GENE), precipitated with ethanol, and dissolved in TE.

The amount of *NINJ2* DNA fragment in the purified input and ChIP-enriched DNA was quantified by qPCR using *NINJ2* primers designed with Primer3Plus. The abundance of *NINJ2* DNA in the ChIP-enriched DNA was normalized by dividing it by the abundance in the input DNA. The percentage input was calculated using the following formula: % Input=100×2^[Ct(Input)−log2(Dilution factor)−Ct(ChIP)]^. To assess the background signal and confirm the specificity of FLAG-AID binding, cells transfected with an empty vector (control#3) were used as negative controls. qPCR was performed using a total reaction volume of 20 µl and an annealing temperature of 60°C. Primer sequences and PCR conditions are listed in Table S4.

### Methylation assay

Extraction of gDNA to determine the methylation status of DLD-1 cells stably expressing an empty vector (control) or AID vector (AID-OP) was performed using a DNeasy Blood and Tissue kit (cat#69504, Qiagen). For bisulfite conversion, 1 µg of gDNA was utilized with the protocol of an EZ DNA methylation kit (cat#D5001, Zymo Research, Irvine, CA, USA). In addition, an EpiTect PCR Control DNA Set (cat#59,695, QIAGEN, Hilden, Germany) was used as a control for methylated and unmethylated DNA. Methylation status of *NINJ2* was then assessed by COBRA and bisulfite sequencing (BSP). *NINJ2* COBRA and BSP common primers were 5′-GYGGTTTATAAATTAGGGAGGGGAGAAGG-3′ (forward) and 5′-ACACCCRATAAAATCAAAAAAAATATCATC-3′ (reverse), which were designed using the Bisulfite Primer Seeker (ZYMO RESEARCH, Irvine, CA, USA). In addition, PCR amplification was performed with Ex Taq HS (cat#RR006A, TaKaRa) for 40 cycles at an annealing temperature of 60°C. After digesting PCR products with *Eco*RI (cat#1040A, TaKaRa) or *Hin*cII (*Hin*dII, cat#1059A, TaKaRa) at 37°C for 5 h, they were separated by electrophoresis on a 2% Tris-acetate-EDTA (TAE) agarose gel and were detected with ProteinSimple SA-1000 (Santa Clara, CA, USA). COBRA was repeated at least three times.

To confirm the COBRA results, PCR amplification was performed under the same PCR conditions (40 cycles at an annealing temperature of 60°C) using the same primers employed in the COBRA analysis (Table S5). PCR products were gel-purified and cloned into the T/A cloning vector pGEM-T Easy (cat#A1360, Promega, Madison, WI, USA). At least five subclones were independently isolated and validated with DNA sequencing performed by Eurofins Genomics (Okada et al., 2013). We analyzed CpG methylation status using a web-based bisulfite sequencing analysis tool called Quantification tool for Methylation Analysis (QUMA, RIKEN, Kobe, Japan).

### Mice and treatment with DSS

C57BL/6J female mice (9-12 weeks of age) were purchased from Charles River Laboratories, Inc. (Tokyo, Japan), and *Aid*-Cre knock-in mice (stock#007770) were purchased from Jackson Laboratory (Bar Harbor, ME, USA). Breeding was conducted in a specific pathogen-free (SPF) room. *Aid*-Cre knock-in mice were produced by replacing exon 1 of the endogenous *Aid* gene with a Cre cassette; when mice were homozygous for the knock-in allele, they exhibited an AID-deficient (AID-KO) phenotype. In this study, homozygous *Aid*-Cre knock-in mice were utilized as AID-KO mice. All animal experiment protocols were approved by the Animal Experiment Review Committee of Kurume University (#2024-063 and #2024-064). Every effort was made to minimize the number of animals used and animal suffering.

Mice were given 4% DSS (colitis grade, MW: 36,000-50,000 Da; cat#9011-18-1, Lot#M7141, MP Biomedicals, Santa Ana, CA, USA) in drinking water for 4 days to induce colitis, followed by normal drinking water. For the epithelial regeneration model (Fig. 6), mice were administered 4% DSS. To minimize inter-individual variability in the initial injury severity, mice exhibiting a 10-20% body weight loss on day 6 were selected for subsequent analysis, following established protocols (Wirtz et al., 2007). Mice falling outside this range or reaching humane endpoints (>25% weight loss) were excluded. For the mild colitis model (Fig. S4), mice were administered 2% DSS. In this model, all DSS-treated mice were included in the analysis, as they exhibited consistent mild colitis. On day 6 (2 days after switching to normal drinking water), mice were euthanized for gene expression analysis to compare mRNA levels between WT and AID-KO mice. Mice were administered 4% or 2% DSS in drinking water for 4 days to induce colitis, followed by normal drinking water until day 13. Body weight, diarrhea, and bleeding of mice were monitored daily, and the scoring of the DAI was performed in a blinded manner. The DAI is the average of the total score for body weight, rectal bleeding, and stool consistency (Cooper et al., 1993).

### Isolation of colonic crypts and total RNA extraction

Colonic crypts isolated from WT and AID-KO mice during the recovery phase from acute intestinal injury were purified using 30 mM EDTA perfusion (Mizoguchi et al., 2002). Total RNA was extracted from colonic crypts using TRIzol reagent (Thermo Fisher Scientific Inc.) and an SV Total RNA Isolation System (Promega, Madison, WI, USA) according to the manufacturer's protocol. Total RNA samples were quantified using a NanoDrop ND-2000 spectrophotometer (cat#ND-2000, Thermo Fisher Scientific Inc.).

### Western blotting and subcellular fractionation

Cells were lysed in lysis buffer [20 mM Tris-HCl (pH 7.5), 150 mM NaCl, 10 mM MgCl_2_, 0.5% Nonidet P-40] containing 2 mM phenylmethylsulfonyl fluoride (PMSF, cat#329-98-6, Sigma), PIC (cat#04080-11, Nacalai Tesque), and phosphatase inhibitor cocktail (cat#07575-51, Nacalai Tesque). Lysate was allowed to stand on ice for 20 min and then centrifuged at 10,000 ***g*** for 20 min at 4°C. Supernatant was collected, and the whole cell lysate (WCL) was sonicated (Handy Sonic, UR-20P, TOMY SEIKO). A total of 40 µg of protein was separated on 5-20% gradient SDS-PAGE gels (cat#197-15011, Fujifilm) and subsequently transferred onto nitrocellulose membranes (cat#1704158, Bio-Rad) for western blotting using a Trans-Blot Turbo system (cat#1704150J2, Bio-Rad).

Nuclear and cytoplasmic fractions were isolated from empty vectors (control#3) or 3FLAG-AID stable expression (AID-FLAG#1) DLD-1 cells using a Subcellular Protein Fractionation Kit (Pierce), according to the manufacturer's protocol (Fig. S3B).

Primary antibodies used for western blotting were as follows: anti-AID (1:1000, rat, cat#4959, EK2 5G9, Cell Signaling Technology), anti-α-Tubulin (1:1000, mouse, cat#SC-32293, DM1A, Santa Cruz), anti-DYKDDDDK tag (1:2000, mouse, cat#014-22383, 1E6, Fujifilm), and anti-H2B (1:20,000, rabbit, cat#ab52599, EP957Y, Abcam, Cambridge, UK). Anti-mouse, anti-rat and anti-rabbit IgG antibodies labeled with either CF680 or CF770 fluorescent dyes (cat#20077, cat#20069 and cat#20067, Biotium, Hayward, CA, USA) were diluted 1:5000 for use in detection. These antibodies were diluted in Blocking One (Nacalai Tesque), and after the antibody reaction, nitrocellulose membranes (cat#1704158, Bio-Rad) were washed four times with PBS containing 0.1% Tween-20 (Nacalai Tesque). Fluorescence on the membrane was detected using an Odyssey instrument (LI-COR, Lincoln, NE, USA).

### Ethics statement

All animal experiments were approved by the Ethics Committee of Kurume University School of Medicine (2024-063). Approval was also obtained from the Safety Committee for Genetic Modification Experiments (27-020).

### Statistics

Statistical analyses were performed using the nonparametric Mann-Whitney *U* test or Pearson correlation analysis with GraphPad Prism 7.0e (https://www.graphpad.com/scientific-software/prism/). For comparisons among three or more groups, one-way ANOVA followed by Tukey's multiple-comparison test was performed. In the figures, asterisks denote statistical significance, indicating *P<0.05, **P<0.01, ***P<0.001, and ****P<0.0001. Values in figures are presented as means and standard deviations (s.d.). *P*-values <0.05 were considered statistically significant.

## Supplementary Material



10.1242/biolopen.062001_sup1 Supplementary information

Dataset 1.

Dataset 2.
