# Supplementary material for: Activation-induced cytidine deaminase promotes DNA demethylation and expression of the Ninjurin-2 gene
Source: Biol Open. 2026 Jun 12;15(6):bio062001. doi: 10.1242/bio.062001 (PMC13312926; doi:10.1242/bio.062001)
Supplement: Supplementary information [file biolopen-15-062001-s1.pdf]

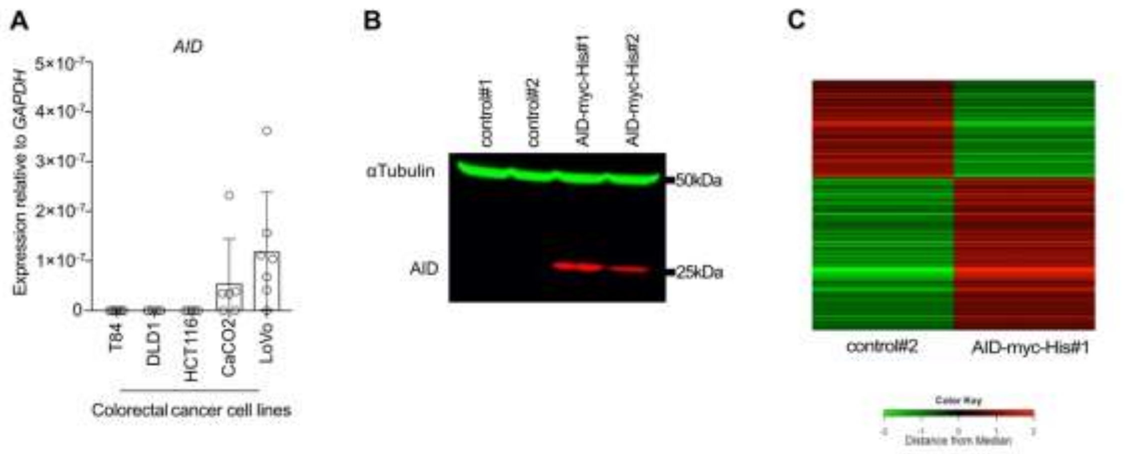

Fig. S1. Generation of AID-overexpressing colorectal cancer cell lines and comprehensive analysis of gene expression by DNA microarray. (A) *AID* expression in representative **colorectal cancer** (CRC) cell lines (T84, DLD-1, HCT116, Caco2, and LoVo) was assessed with RT-qPCR. RT-qPCR measurements were performed in technical duplicates or triplicates. The number of independent cell cultures from the left is: n=7, 6, 5, 6, or 7. Error bars represent means ± SDs. Among these cell lines, DLD-1 cells showed no detectable endogenous *AID* expression and were therefore selected for subsequent AID overexpression experiments. **(B)** Expression levels of AID protein were assessed by Western blotting using empty vectors (control) or stably expressing AID (AID-myc-His) transfected DLD-1 cells. α-Tubulin (green) was used as a loading control, and AID protein is shown in red. Data are representative of more than three independent experiments. **(C)** For microarray analysis, stable AID- or empty vector-expressing DLD-1 cells (AID-myc-His#1 and control#2) were generated. A heat map of genes with Z-score changes of ≥2 in AID-myc-His#1 compared with control#2 cells is shown in the diagram. Genes that increased in AID-myc-His#1 are shown in red and those that decreased are shown in green.

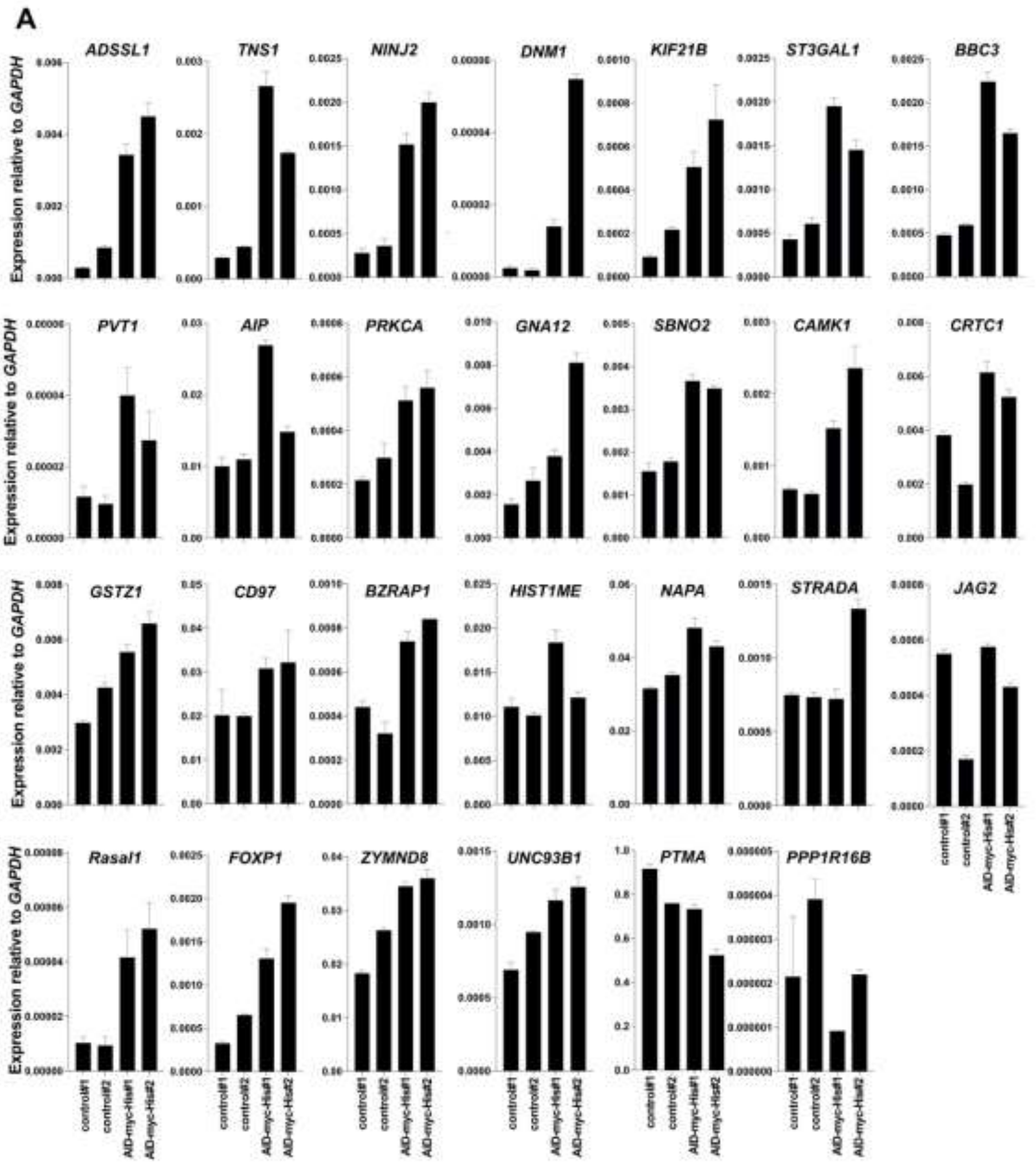

Fig. S2. Expression levels of 33 genes predicted to bind AID. (A) Expression levels of 27 genes were assessed using control and AID-OP cells. RT-qPCR was performed using cDNA generated from independent cell cultures (n=2-4). Each measurement was conducted in a single well. Levels of 6 genes, *ITM2C*, *NKD2*, *RPL7A*, *TREX1*, *KLF3*, and *PRR7*, could not be quantified due to amplification failure in qPCR. Sample labels are shown below each column.

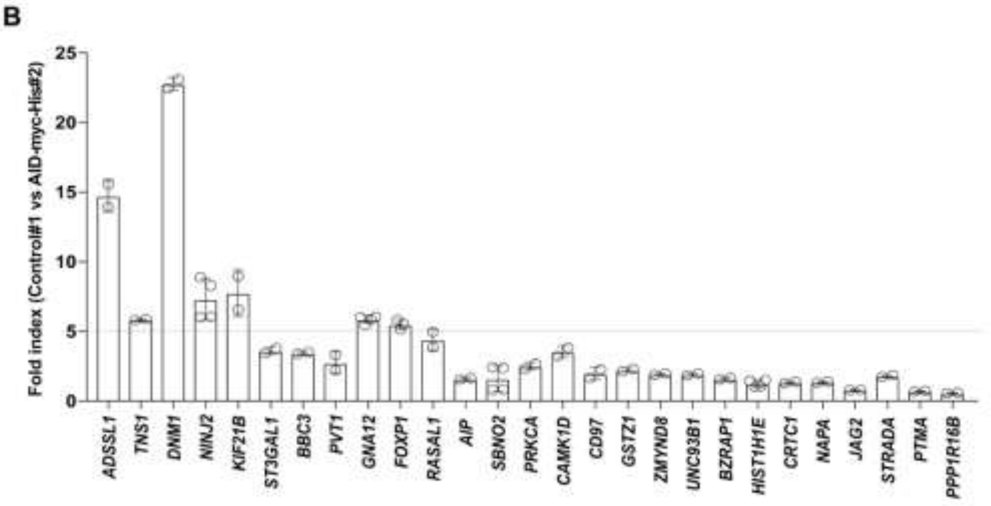

Fig. S3. Expression levels of 33 genes predicted to bind AID. (B) Relative expression of each gene in AID-myc-His#2 cells is shown in comparison to control#1 cells. RT-qPCR was performed using cDNA generated from independent cell cultures. Each measurement was conducted in a single well. The number of biological replicates was as follows: n = 4 for *NINJ2*, *GNA12*, *SBNO2*, and *HIST1H1E*, n = 3 for *FOXP1*, and n = 2 for all other genes. Individual data points are plotted as open circles, and bar graphs represent the mean ± SD.

Biology Open • Supplementary information

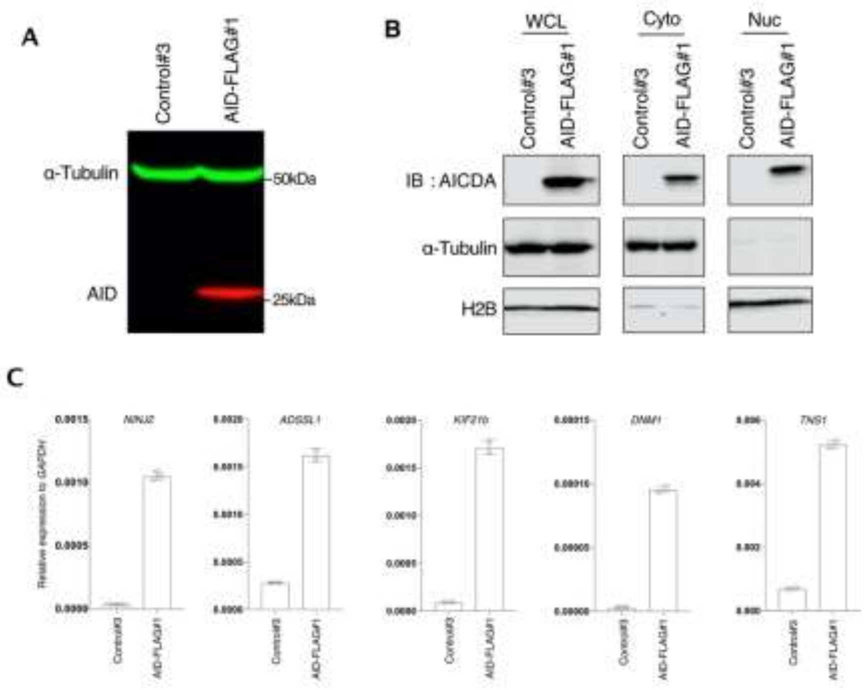

Fig. S4. Expression and localization of AID in AID-FLAG-overexpressing cells and analysis of AID target genes. (A) Expression levels of AID proteins in the DLD-1 cell line stably expressing an empty vector (control#3) or AID-3FLAG (AID-FLAG#1) were assessed by western blotting. Green and red bands indicate α-Tubulin and AID, respectively. **(B)** Localization of AID in control#3 and AID-FLAG#1 cells was confirmed by western blotting after subcellular fractionation. **(C)** Using the control#3 and stable AID-3FLAG expression cell lines, which were newly generated to perform ChIP, five genes (*NINJ2*, *ADSSL1*, *KIF21b*, *DNM1* and *TNS1*) were assessed to determine whether they were upregulated by AID expression. RT-qPCR measurements were performed in duplicate wells. The bar graphs represent the mean ± SD of results from n = 2 or 3 independent cell cultures. WCL; whole cell lysate, Cyto; Cytoplasm fraction, Nuc; Nuclear fraction.

Biology Open • Supplementary information

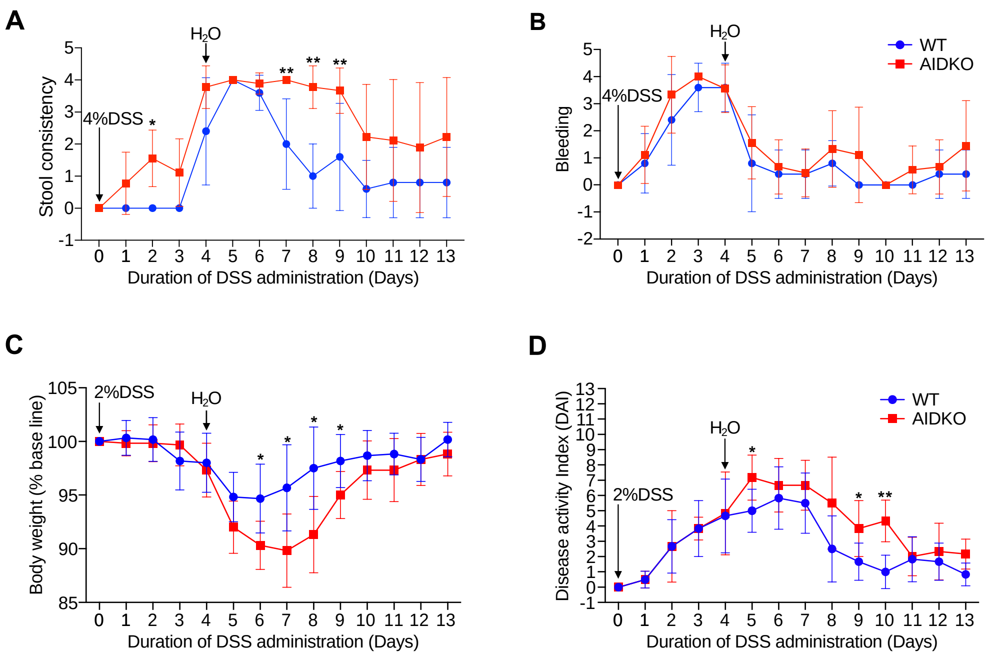

Fig. S5. Assessment of Disease Activity Index (DAI) in WT and AID-KO mice with DSS acute colitis. DAI index for WT and AID-KO mice with 4% DSS acute colitis is the total score for body weight, **(A)** stool consistency, and **(B)** rectal bleeding (n=5, 9). **(C)** 2% DSS was administered to WT and AID-KO mice in drinking water for 4 days to induce colitis, followed by normal drinking water until day 13 (n=6 each). **(D)** Body weight, diarrhea, and bleeding of WT and AID-KO mice were monitored daily, and the disease activity index (DAI) was evaluated (n=6 each). The DAI is calculated by scoring body weight, bloody stools, and diarrhea (**Table S4),** divided by 3 to derive the mean value. *n* indicates the number of individual mice used for each group. Blue and red lines indicate WT and AID-KO mice, respectively. Statistical significance was determined using the nonparametric Mann-Whitney U test. KO, knockout; WT, wild type. *, $P < 0.05$, **, $P < 0.01$. Error bars represent the mean ± SD.

Biology Open • Supplementary information

Table S1. Cloning primer for *AID*.

Name	sequence(5'-3')	Restrict enzyme
AID cloning primer Forward	ACGC**GGATCCGCTAGC**ACCACTATGGACAGCCTCTTGATGAACC	① *NheI* ,② *BamHI*
AID cloning primer Reverse	TCCC**AAGCTTGGGCCC**AAGTCCCAAAGTACGAAATGCG	① *HIndIII* ,② *APaI*
AID-3XFLAG cloning primer Forward	gccGCTAGCATGGGTGTGAAGGCGTCTC	*NheI*
AID-3XFLAG cloning primer Reverse	gccAAGCTTgacTCACTTGTCGTCGTCGTCCTTGTAGTCGATGTCGTGGTC	*HindIII*

Table S2. The primer pair for colony direct PCR and BigDye sequence.

Name	Tm(℃)	sequences (5'-3')
CMV-Forward	55	CGCAAATGGGCGGTAGGCGTG
EGFP-N-Reverse		CGTCGCCGTCCAGCTCGACCAG
T7-Forward	55	TAATACGACTCACTATAGGG
BGH-Reverse		GCTGGCAACTAGAAGGCACAG
M13-RV	55	CAGGAAACAGCTATGAC

Table S3. Quantitative PCR primers.

#	gene	Sequence(5'-3')	Sequence(3'-5')	Amplicon size(bp)	Tm (°C)	species
1	***ADSSL1***	CGGGACCTACCCCTTTGTG	CACGCCATACACGTCACCT	100	60	human
2	***TNS1***	CGTGCAGGGAGTCTTTCTTC	TACGACAACTTCAGTGGGCA	115	60	human
3	***DNM1***	CGGTGAATTTCTTTCCCTTG	GCTCGAGAATTTCGTAGGCA	137	60	human
4	***NINJ2***	TTGCACGGCTGAACCTGAAT	CCTGTTTTATGTGCCCCGAA	133	60	human
5	***KIF21B***	ACCTATGACTTTGTCTTCGACCT	CAGCACCGTGGCATTATAGC	108	60	human
6	***ST3GAL1***	GGAGGACGACACCTACCGAT	CCACCGACCTCTTCTCCAG	134	60	human
7	***BBC3***	CTGGGTAAGGGCAGGAGTC	GACGACCTCAACGCACAGT	113	60	human
8	***PVT1***	TGAGAACTGTCCTTACGTGACC	AGAGCACCAAGACTGGCTCT	74	60	human
9	***GNA12***	TGTGTTGAGACCGTGTGTGT	AGCAGCCTCTGAAGTCATCT	146	60	human
10	***FOXP1***	CAGATATTGCGCAGAACCAA	GCAAACATTCGTGTGAACCA	151	60	human
11	***RASAL1***	TGGATTTCTCTTCTTGCGATTCT	TGTTGGTCCCGAAGGTCAA	72	60	human
12	***AIP***	GGTAGTGGAACGTGGCCTT	AGGAGGATGGCGGATATCAT	121	60	human
13	***SBNO2***	ACTCCCTGTCGGACATCGT	GAACAGCTTATCGTGGGTGGA	113	60	human
14	***PRKCA***	TCAGTGTCGGGTCCCTTATC	CGACTTCATCTGGGGGTTT	126	60	human
15	***CAMK1D***	CTTCAGCGCCTTCTTAGGG	CGAGTTCAAAGAGACCCTCG	112	60	human
16	***CD97***	GGAGTCCTGGGTTTCAGCTC	TGTCCCACTCACTCTTTCCC	116	60	human
17	***GSTZ1***	GTGTAGGAGACGAGGTGACC	TCAGTGGGTGTATCTGGCTG	178	60	human
18	***ZMYND8***	CGAGACCCAGAGTAAAGCCAT	GATGTATTCCGCATAGTCAGGG	151	60	human
19	***UNC93B1***	GACAAAGAGGGCGTAGATGC	ACAGCAAAATGCTGATGGG	128	60	human
20	***BZRAP1***	AGGGAGGTCAGGAGGTCATC	TATGGCAACATGGATGAGGA	116	60	human
21	***HIST1H1E***	AGAGCCTTTGGGTTGTGTTG	CGAAGGCCAAAGCAGTTAAA	145	60	human
22	***CRTC1***	TCAGTGGACAAACACGGACG	CAGGGCGGAGTCAGAATTG	108	60	human
23	***NAPA***	TGCCCACTACGAGCAGTCT	CATTGGTCCCCACCTGTTCG	149	60	human
24	***JAG2***	CTCCTCATTCGGGGTGGTAT	GTCGTCATCCCCTTCCAGT	93	60	human
25	***STRADA***	GCGAATCAGGACCAATGATGC	CAGTGAGCAGCTCGTAACACC	107	60	human
26	***PTMA***	CTCCTCATTCGGGGTGGTAT	GTCGTCATCCCCTTCCAGT	93	60	human
27	***PPP1R16B***	CGTCCTCATTGCACAAATCA	GCCGCAAGAAAGTGTCCTT	123	60	human
28	***GAPDH***	TTGAGGTCAATGAAGGGGTC	GAAGGTGAAGGTCGGAGTCA	117	60	human
29	***AID***	AAATGTCCGCTGGGCTAAGG	GGAGGAAGAGCAATTCCACGT	140	60	human
30	***ADSSL1***	ACCAAGGCCGTGTCATTCAT	AAGTCGAACACAAGGTGAGC	146	60	Mouse
31	***TNS1***	CTGGTGTATGTCACCGAACG	GTTCAGAGAGGTTGAATAGCAGG	145	60	Mouse
32	***DNM1***	GAACTGCGAAGGGAGATCAG	GGTCACAATTCGCTCCATCT	171	60	Mouse
33	***NINJ2***	AGACGTGGCGCTCTTTATGT	TCAGGATGGCGATGAACACA	158	60	Mouse
34	***KIF21B***	AACAACCGGGTCACACAACT	TTTGTTCGCAGCTCCTCGAT	128	60	Mouse
35	***HPRT1***	ACAGGCCAGACTTTGTTGGA	GCAGATTCAACTTGCGCTCA	159	60	Mouse

Table S4. ChIP-qPCR primers.

gene	Sequence(5'-3')	Amplicon size(bp)	Tm (°C)
NINJ2-F	AGGGAAGAAAGACCCGATTC	112	60
NINJ2-R	TCCAGCCCTAAAATGACGAC		

Table S5. BSP and COBRA primers

gene	Sequence(5'-3')	Amplicon size(bp)	Tm (°C)
NINJ2-F	GYGGTTTATAAATTAGGGAGGGGAGAAGG	439	60
NINJ2-R	ACACCCRATAAAATCAAAAAAAATATCATC		

Table S6. Disease activity index (DAI) score.

Score	Body weight decrease(%)	Stool consistency	Bleeding
0	< 1	Normal	Normal
1	1-5		
2	5-10	Loose stools	
3	10-15		
4	>20	Diarrhea	Gross bleeding

Dataset 1.

Available for download at
https://journals.biologists.com/bio/article-lookup/doi/10.1242/bio.062001#supplementary-data

Dataset 2.

Available for download at
https://journals.biologists.com/bio/article-lookup/doi/10.1242/bio.062001#supplementary-data

Biology Open • Supplementary information
