# Supplementary material for: Activation-induced cytidine deaminase promotes DNA demethylation and expression of the Ninjurin-2 gene
Source: Biol Open. 2026 Jun 12;15(6):bio062001. doi: 10.1242/bio.062001 (PMC13312926; doi:10.1242/bio.062001)
Supplement: Supplementary file 1 [file biolopen-15-062001_review_history.pdf]

Activation-induced cytidine deaminase promotes DNA demethylation and expression of the Ninjurin-2 gene

Yusuke Toyoda, Shigeaki Saitoh and Toshiyuki Okada
10.1242/bio.062001

Editor: Christopher A. Maher

Review timeline

Original submission:	26 March 2025
Editorial decision:	21 April 2025
First revision received:	22 May 2025
Editorial decision:	11 June 2025
Second revision received:	29 August 2025
Editorial decision:	30 October 2025
Third revision received:	29 January 2026
Editorial decision:	6 February 2026
Fourth revision received:	6 May 2026
Accepted:	12 May 2026

Original submission

First decision letter

MS ID#: bio.062001

MS Title: Activation-induced cytidine deaminase promotes DNA demethylation and expression of the Ninjurin-2 gene

Authors: Toshiyuki Okada, Yusuke Toyoda and Shigeaki Saitoh

I have now reached a decision on the above manuscript.

The reviewer reports are shown at the bottom of this email or can be accessed, together with a copy of this decision letter, by going to:

As you will see, the reviewers raised a number of substantial criticisms that prevent me from accepting the paper at this stage.

They suggest, however, that a revised version might prove acceptable, if you can address their concerns. If you think that you can deal satisfactorily with the criticisms on revision, I would be pleased to see a revised manuscript. We would then return it to the reviewers.

At this stage, we also ask you to ensure your manuscript complies with our formatting guidelines. Provided you are able to fully address the referees' comments, we are positive about publication of your paper (we accept over 95% of revision submissions) and therefore hope you won't mind any extra work involved in reformatting your manuscript at this point.

Please ensure that you clearly highlight all changes made in the revised manuscript. Please avoid using 'Tracked changes' in Word files as these are lost in PDF conversion.

© 2026. Published by The Company of Biologists under the terms of the Creative Commons Attribution License (https://creativecommons.org/licenses/by/4.0/).

I should be grateful if you would also provide a point-by-point response detailing how you have dealt with the points raised by the reviewers in the 'Response to Reviewers' box. Please attend to all of the reviewers' comments. If you do not agree with any of their criticisms or suggestions please explain clearly why this is so.

Reviewer 1

Comments for the author

The manuscript entitled "Activation-induced cytidine deaminase promotes DNA demethylation and expression of the Ninjurin-2 gene" provides compelling evidence that AID, beyond its established role in immunoglobulin diversification, facilitates DNA demethylation and transcriptional activation of NINJ2 in colonic epithelial cells during inflammation. The authors employed a comprehensive combination of in vitro (CRC cell lines) and in vivo (DSS-induced colitis in AID-KO mice) models, along with molecular assays (ChIP, BSP, COBRA, and siRNA) to substantiate their primary hypothesis. This study is innovative and relevant to the study of inflammation-related epithelial repair and epigenetic regulation.

Strengths: This subject is of considerable biological and clinical interest, particularly in the context of IBD. The experimental design was coherent and the methodologies were appropriate and detailed. The data robustly support these findings, and AID's role of AID in DNA demethylation is clearly demonstrated.

Suggestions for Improvement: Although the regulatory effect of AID on NINJ2 is well documented, further functional validation of NINJ2 (e.g., knockdown studies examining cell proliferation, survival, or necroptosis) would enhance the mechanistic link to epithelial regeneration. The use of systemic AID-knockout mice, while valid, may introduce potential confounding factors owing to alterations in the immune system and microbiota. Although this limitation is acknowledged, it should be discussed more prominently, particularly regarding its impact on interpreting the in vivo colitis recovery results. The authors demonstrated a locus-specific demethylation event at the NINJ2. It would be beneficial to discuss whether this indicates a broader epigenetic program regulated by AID or whether it is unique to this locus.

Minor Points: Some p-values (e.g., $P = 0.05$) were borderline; reporting effect sizes and clarifying biological replicates would be useful. Figure references (for example Fig. S1A and S3C) should be clearly explained in the figure legends to ensure that they are self-contained. The manuscript is generally well written. Light language editing would further enhance the clarity.

Conclusion: This well-executed and insightful study broadens our understanding of AID's functions of AID beyond B cells. I recommend minor revisions to address the comments above and to improve the clarity and interpretation of the results.

Reviewer 2

Comments for the author

The manuscript presents compelling data demonstrating that inflammation-induced expression of AID promotes NINJ2 transcription by binding to the promoter/enhancer region and modulating DNA methylation. The study utilizes both DLD-1 cells overexpressing AID and AID knockout mice, and overall, the manuscript is well written and accessible even to non-expert readers.

While the data generally support the authors' conclusions, a few points merit further clarification:

In Figure 1, AID-OP#1 and AID-OP#2 were used, and Supplementary Figure S1B shows some variability in AID expression levels between these two lines. However, the methylation status of the NINJ2 gene was assessed using AID-OP#3, and the expression level of AID in this line was not presented. Could the authors please clarify the rationale for choosing AID-OP#3 for the methylation analysis and provide data on AID expression in this line for completeness?

© 2026. Published by The Company of Biologists under the terms of the Creative Commons Attribution License (https://creativecommons.org/licenses/by/4.0/).

Have the authors considered analyzing the methylation status of NINJ2 in AID knockout cells to strengthen the conclusion that AID directly contributes to the demethylation of the NINJ2 gene? Given the complexity of transcriptional regulation, it would be helpful to address the possibility that other factors may also contribute to NINJ2 expression under inflammatory conditions.

It would be valuable if the authors could elaborate on how AID expression is induced during inflammation. Specifically, which upstream signaling pathways or inflammatory mediators are involved in regulating AID expression?

Reviewer's Responses to Questions

Experimental quality
Does each figure have the proper controls?
If 'No', please indicate reasons in Comments for Author box below.
Reviewer #1:
- Yes

Reviewer #2:
- Yes

Were the data analyzed using appropriate statistical tests?
If 'No', please indicate reasons in Comments for Author box below.
Reviewer #1:
- Yes

Reviewer #2:
- Yes

Reproducibility
Were experiments performed using adequate number of biological replicates?
If 'No', please indicate reasons in Comments for Author box below.
Reviewer #1:
- Yes

Reviewer #2:
- Yes

Does the methods section provide sufficient detail to permit reproducibility?
If 'No', please indicate reasons in Comments for Author box below.
Reviewer #1:
- Yes

Reviewer #2:
- Yes

Completeness
Are the manuscript's conclusions supported by the data?
If 'No', please indicate reasons in Comments for Author box below.
Reviewer #1:
- Yes

Reviewer #2:
- Yes

Scholarship
Do the authors cite and discuss the merits of data that would argue for and against their conclusion?
If 'No', please indicate reasons in Comments for Author box below.
Reviewer #1:
- Yes

© 2026. Published by The Company of Biologists under the terms of the Creative Commons Attribution License (https://creativecommons.org/licenses/by/4.0/).

Reviewer #2:
- Yes

Does the manuscript title & abstract accurately reflect the contents of the manuscript, without hyperbole?
If 'No', please indicate reasons in Comments for Author box below.
Reviewer #1:
- Yes

Reviewer #2:
- Yes

First revision

Author response to reviewers' comments

We sincerely thank the reviewers for their thoughtful and constructive comments on our manuscript. We have revised the manuscript accordingly and provide detailed responses to each comment below. Changes in the revised manuscript are indicated in red text or yellow highlighting in the revised manuscript and supplementary information.

Response to Reviewer 1:

Comment 1: Suggestions for Improvement: Although the regulatory effect of AID on NINJ2 is well documented, further functional validation of NINJ2 (e.g., knockdown studies examining cell proliferation, survival, or necroptosis) would enhance the mechanistic link to epithelial regeneration.
Response: We thank the reviewer for this insightful suggestion regarding further functional validation of NINJ2. We fully agree that knockdown studies would strengthen the mechanistic connection between NINJ2 and epithelial regeneration. However, as the primary aim of this study was to investigate epigenetic regulation of NINJ2 by AID, we did not include functional assays in the current work.
Nevertheless, in the revised Discussion **(Page 13, Lines 290-291),** we have noted that this represents an important direction for future research.

Comment 2: The use of systemic AID-knockout mice, while valid, may introduce potential confounding factors owing to alterations in the immune system and microbiota. Although this limitation is acknowledged, it should be discussed more prominently, particularly regarding its impact on interpreting the in vivo colitis recovery results.
Response: We appreciate this valuable comment. We have revised the limitations section to more clearly emphasize the potential impact of using systemic AID-knockout mice, particularly in interpreting colitis recovery outcomes. Specifically, we now highlight how systemic alterations, such as reduced populations of butyrate-producing bacteria and changes in bile acid metabolism, may affect epithelial regeneration and immune regulation during the recovery phase **(Page 13, Lines 296-304)**. We believe this revision provides better context for our in vivo findings by acknowledging the broader limitations of the model employed.

Comment 3: The authors demonstrated a locus-specific demethylation event at the NINJ2. It would be beneficial to discuss whether this indicates a broader epigenetic program regulated by AID or whether it is unique to this locus.
Response: This is an excellent suggestion. Accordingly, in the revised manuscript we have added a discussion of the potential scope of AID's epigenetic effects. Specifically, we noted that while AID can induce somatic hypermutation in immunoglobulin genes and hotspot mutations in genes such as TP53 (Takai et al., 2012b), its demethylating activity may similarly be restricted to specific cytosine residues.

© 2026. Published by The Company of Biologists under the terms of the Creative Commons Attribution License (https://creativecommons.org/licenses/by/4.0/).

We also discussed the possibility that broader demethylation patterns may occur indirectly through involvement of additional demethylating enzymes or co-factors **(Page 11, Lines 243-246)**.

Comment 4 (Minor): Some p-values (e.g., P = 0.05) were borderline; reporting effect sizes and clarifying biological replicates would be useful.
Response: We thank the reviewer for this helpful comment. We have clarified the number of biological replicates for each relevant experiment by specifying individual replicate counts in the figure legends **(Page 29 Line 686; Page 31, Lines 721-723)** and in the supplementary figure legends **(Supplementary information: Pages 1-2, 5)**.

Comment 5(Minor): Figure references (for example Fig. S1A and S3C) should be clearly explained in the figure legends to ensure that they are self-contained. The manuscript is generally well written. ***Light language editing would further enhance the clarity***.
Response: We thank the reviewer for the careful reading and helpful comment. We have revised the figure legends for **Fig. S1** and **Fig. S3** to include more detailed explanations **(Supplementary information: Pages 1, 4)**. Specifically, in **Fig. S3C**, we modified the number of genes analyzed (five genes) and revised the experimental description to indicate that at least two independent experiments were performed. Additionally, to avoid confusion regarding DSS concentrations in **Fig. S4**, we added labels for "4% DSS" and "H_2O" directly into the graphs in **Fig. S4A and S4B (Supplementary information: Page 5)**.
The revised figure legends and manuscript text have also been edited by an experienced professional editor, who is a native English (U.S.) speaker. We hope these revisions address the concern and improve the overall quality of the manuscript. Changes are indicated in red in the supplementary information.

Response to Reviewer 2:
Comment 1: In Figure 1, AID-OP#1 and AID-OP#2 were used, and Supplementary Figure S1B shows some variability in AID expression levels between these two lines. However, the methylation status of the NINJ2 gene was assessed using AID-OP#3, and the expression level of AID in this line was not presented. Could the authors please clarify the rationale for choosing AID-OP#3 for the methylation analysis and provide data on AID expression in this line for completeness?
Response: We thank the reviewer for this valuable comment, and we apologize for any confusion. Expression of AID in the AID-OP#3 cell line (now referred to as AID-FLAG#1) was confirmed by Western blotting, as shown in **Figure S3A**, with α-Tubulin as a loading control. As ChIP analysis is highly sensitive, we sought to avoid potential issues with non-specific antibody binding due to cross-reactivity with endogenous c-Myc. Therefore, since anti-FLAG antibodies perform well in ChIP assays, we generated a FLAG-tagged AID-expressing cell line (AID-FLAG#1) and used it for both ChIP-qPCR and subsequent DNA methylation analysis. **(Pages 7-8, Lines 168-171)**
For greater clarity, we have renamed the cell lines. AID-OP#1 and AID-OP#2 were changed to AID-Myc- His#1 and AID-Myc-His#2, respectively, and AID-OP#3 was changed to AID-FLAG#1. These changes are highlighted in yellow in the revised manuscript **(Page 5, Lines 105-107, 110, 118-119; Page 6, Lines 125-126; Page 7, Lines 153-155, 158-159; Page 8, Lines 173, 177, 179, 187-188; Page 9, Lines 196**, 199; Page 15, Line 355; Page 20, Line 463; Page 29, Lines 674, 679-680, 692-693; Page 30, Lines 699, 708, 712).

Comment 2*: Have the authors considered analyzing the methylation status of NINJ2 in AID knockout cells to strengthen the conclusion that AID directly contributes to the demethylation of the NINJ2 gene? Given the complexity of transcriptional regulation, it would be helpful to address the possibility that other factors may also contribute to NINJ2 expression under inflammatory conditions.*
Response: We appreciate this insightful comment. We agree that analyzing methylation status of NINJ2 in AID-deficient cells would further support the direct involvement of AID in its demethylation. However, because DNA methylation changes occur at the single-cell level, bulk *in vivo* analysis tends to produce a mosaic pattern, making accurate locus-specific evaluation difficult. Therefore, we did not perform in vivo methylation analysis in AID-deficient mice. We also agree that other factors may contribute to NINJ2 expression under inflammatory conditions. To address this point, we have added the following statement **(Pages 12-13, Lines 287-290)**: " Moreover, given that mutations in the C/EBP beta binding site in the NINJ2 promoter region in humans, which is associated with inflammatory responses, lead to reduced NINJ2 mRNA expression (Wang et al.,

© 2026. Published by The Company of Biologists under the terms of the Creative Commons Attribution License (https://creativecommons.org/licenses/by/4.0/).

2021), it is possible that other factors independent of AID also contribute to regulation of NINJ2 under inflammatory conditions."

Comment 3: *It would be valuable if the authors could elaborate on how AID expression is induced during inflammation. Specifically, which upstream signaling pathways or inflammatory mediators are involved in regulating AID expression?*
Response: We are grateful for this valuable suggestion. To address this question, we have added the following statement **(Page 4, Lines 82-84)**: "Accordingly, AID expression in various epithelial cells is induced by NF-κB activation through TNF-α stimulation and is further enhanced by the Th2 cytokines L4, IL-13, and IL-21 (Araki et al., 2019; Endo et al., 2008; Watashi et al., 2013)."

Second decision letter

MS ID#: bio.062001R1

MS Title: Activation-induced cytidine deaminase promotes DNA demethylation and expression of the Ninjurin-2 gene

Authors: Toshiyuki Okada, Yusuke Toyoda and Shigeaki Saitoh

I have now reached a decision on the above manuscript.

The reviewer reports are shown at the bottom of this email or can be accessed, together with a copy of this decision letter, by going to:

As you will see, the reviewers gave favourable reports, but raised some critical points that will require amendments to your manuscript. I hope that you will be able to carry these out, because we would like to be able to accept your paper.

At this stage, we also ask you to ensure your manuscript complies with our formatting guidelines - please see our manuscript preparation guidelines for details. Provided you are able to fully address the referees' comments, we are positive about publication of your paper (we accept over 95% of revision submissions) and therefore hope you won't mind any extra work involved in reformatting your manuscript at this point.

Please ensure that you clearly highlight all changes made in the revised manuscript. Please avoid using 'Tracked changes' in Word files as these are lost in PDF conversion.

I should be grateful if you would also provide a point-by-point response detailing how you have dealt with the points raised by the reviewers in the 'Response to Reviewers' box. Please attend to all of the reviewers' comments. If you do not agree with any of their criticisms or suggestions please explain clearly why this is so.

Reviewer 1

Comments for the author

I have read your revised manuscript with great interest. The study investigates the role of activation-induced cytidine deaminase (AID) in promoting DNA demethylation of the NINJ2 promoter/enhancer, which in turn regulates gene expression in colonic epithelial cells (CECs) during inflammation. Overall, this is a well-structured and thorough study that addresses a relevant and interesting question in the field of inflammation and epithelial regeneration. I appreciate the use of both in vitro and in vivo models to support your conclusions.

Below, I provide detailed comments organized according to the journal's review criteria, which I hope you will find helpful in refining your manuscript.

© 2026. Published by The Company of Biologists under the terms of the Creative Commons Attribution License (https://creativecommons.org/licenses/by/4.0/).

Experimental Quality

The experiments appear to be well-controlled. The use of AID-overexpressing (AID-OP) cell lines alongside appropriate empty vector controls, as well as the AID-knockout (KO) mouse model, is appropriate for addressing the study's aims.

I suggest clarifying the statistical methods used for analyzing high-throughput data, particularly regarding multiple testing correction in the microarray analysis (e.g., FDR adjustment). This would strengthen the robustness of the conclusions.

For the ChIP-qPCR and bisulfite sequencing assays, please include more detail on negative controls (e.g., non-transfected cells or IgG controls) to confirm the specificity of the binding and methylation changes observed.

Reproducibility

The number of biological replicates appears to be adequate throughout the manuscript. It would be helpful to explicitly state the number of biological replicates for each key experiment, including cell lines and animal groups.

Consider including key raw data (e.g., qPCR Ct values or ChIP enrichment data) in supplementary materials or as source data files. This would help other researchers assess the rigor of the analyses and facilitate reproducibility.

While the methods section is generally detailed, please ensure that all primers (e.g., for qPCR and bisulfite sequencing) and PCR conditions are included in the manuscript or supplementary tables for full reproducibility.

Completeness

The conclusions are well supported by the data presented. The manuscript effectively shows that AID binds to the NINJ2 promoter/enhancer and reduces DNA methylation, which correlates with increased gene expression.

A limitation acknowledged by the authors is that AID-KO mice exhibit systemic immune alterations that might indirectly affect epithelial regeneration. A discussion on how these systemic effects might influence the interpretation of the results would further strengthen the manuscript.

To more fully establish the causality between NINJ2 expression and tissue regeneration, an experiment using NINJ2 knockdown in the AID-OP model (or in vivo) could be considered in future studies. However, I recognize that this may be beyond the current scope.

Scholarship

The authors cite relevant data that both support and challenge their conclusions, including discussion of AID's known roles in carcinogenesis and inflammation. This balanced discussion is commendable.

The manuscript effectively places the study within the context of the broader field, referencing relevant literature on DNA demethylation mechanisms and the roles of AID.

Other Comments

The title and abstract accurately reflect the content of the manuscript and avoid hyperbole.

Overall, the manuscript is clear and well written, though minor editorial polishing (e.g., consistent gene/protein nomenclature) would further improve readability.

Summary

© 2026. Published by The Company of Biologists under the terms of the Creative Commons Attribution License (https://creativecommons.org/licenses/by/4.0/).

This is a strong study that contributes novel insights into the role of AID in epithelial regeneration through DNA demethylation. Addressing the points above—especially regarding statistical analysis, reproducibility, and discussion of limitations—would enhance the clarity and impact of the manuscript.

Thank you for the opportunity to review this interesting and well-executed study.

Reviewer 2

Comments for the author

The authors have thoroughly addressed all the points and questions raised, and have incorporated the suggested modifications into the figures, figure legends, Results, and Discussion sections of the revised manuscript.

Reviewer's Responses to Questions

Experimental quality
Does each figure have the proper controls?
If 'No', please indicate reasons in Comments for Author box below.
Reviewer #1:
- Yes

Reviewer #2:
- Yes

Were the data analyzed using appropriate statistical tests?
If 'No', please indicate reasons in Comments for Author box below.

Reviewer #1:
- Yes

Reviewer #2:
- Yes

Reproducibility
Were experiments performed using adequate number of biological replicates?
If 'No', please indicate reasons in Comments for Author box below.

Reviewer #1:
- Yes

Reviewer #2:
- Yes

Does the methods section provide sufficient detail to permit reproducibility?
If 'No', please indicate reasons in Comments for Author box below.

Reviewer #1:
- Yes

Reviewer #2:

© 2026. Published by The Company of Biologists under the terms of the Creative Commons Attribution License (https://creativecommons.org/licenses/by/4.0/).

- Yes

Completeness
Are the manuscript's conclusions supported by the data?
If 'No', please indicate reasons in Comments for Author box below.

Reviewer #1:
- Yes

Reviewer #2:
- Yes

Scholarship
Do the authors cite and discuss the merits of data that would argue for and against their conclusion?
If 'No', please indicate reasons in Comments for Author box below.

Reviewer #1:
- Yes

Reviewer #2:
- Yes

Does the manuscript title & abstract accurately reflect the contents of the manuscript, without hyperbole?
If 'No', please indicate reasons in Comments for Author box below.

Reviewer #1:
- Yes

Reviewer #2:
- Yes

Second revision

Author response to reviewers' comments

We thank the reviewer for their thoughtful, constructive comments on our manuscript. While reorganizing the raw data, we re-analyzed our figures and corrected some calculation errors in the qPCR datasets (Figure 1C, Figure 2B, Figures S2A–S2B). The corresponding figures have been updated accordingly. Importantly, these corrections do not affect the overall conclusions of this study. All changes in the manuscript and supplementary information are highlighted in the revised version.

The changes resulting from re-analysis are summarized as follows:
1. Minor corrections were made to GNA12 and NINJ2 in **Figure 1C**, as well as **Figures S2A** and **S2B**. These figures have been replaced.
2. Calculation errors in qPCR data for Kif21b (WT-DSS: 1 site, KO-DSS: 2 sites), Adssl1 (WT-DSS: 1 site), and Dnm1 (WT-DSS: 1 site) were corrected, and **Figure 2B** has been updated accordingly.
3. Re-analysis revealed a significant difference in Adssl1 mRNA expression between WT and KO mice with acute colitis conditions **(Figure 2B) (Pages 6, Lines 142-143)**.
4. The relevant raw data are highlighted in red in the **Supplementary Materials**.

© 2026. Published by The Company of Biologists under the terms of the Creative Commons Attribution License (https://creativecommons.org/licenses/by/4.0/).

Response to Reviewer 1:

***Comment 1**: The experiments appear to be well-controlled. The use of AID-overexpressing (AID-OP) cell lines alongside appropriate empty vector controls, as well as the AID-knockout (KO) mouse model, is appropriate for addressing the study's aims. I suggest clarifying the statistical methods used for analyzing high-throughput data, particularly regarding multiple testing correction in the microarray analysis (e.g., FDR adjustment). This would strengthen the robustness of the conclusions.*

Response: We thank the reviewer for this valuable comment. To address this point, we have added the following statement **(Page16, Lines 371-376)**: "Due to the limitation of having only one sample per group, statistical testing or multiple correction, e.g., FDR, could not be applied. Instead, we used Z-scores, which measure how much a gene's expression ratio deviates from the average. Genes with Z-scores above 2 or below -2 and fold changes ≥1.5 or ≤0.67 were considered significant. To reduce false positives in low-expression areas, Z-scores were calculated within intensity-based segments, following Quackenbush (2002)."

***Comment2**: The number of biological replicates appears to be adequate throughout the manuscript. It would be helpful to explicitly state the number of biological replicates for each key experiment, including cell lines and animal groups. Consider including key raw data (e.g., qPCR Ct values or ChIP enrichment data) in supplementary materials or as source data files. This would help other researchers assess the rigor of the analyses and facilitate reproducibility.*

Response: We thank the reviewer for this helpful suggestion. We have now provided key raw data, including qPCR Ct values **(Figures 1-3)** and ChIP enrichment data **(Figure 4)**, in the **Supplementary Materials** to facilitate assessment of the analyses and reproducibility. We also added the following statement to clarify: "Data supporting findings of this study are available in the **Supplementary Materials**." **(Page22, Lines523).**

***Comment3**: For the ChIP-qPCR and bisulfite sequencing assays, please include more detail on negative controls (e.g., non-transfected cells or IgG controls) to confirm the specificity of the binding and methylation changes observed.*

Response: For ChIP-qPCR **(Figure 4)**, we used Control#3 cells (transfected with empty vector) as a negative control, which are not expected to be immunoprecipitated by the anti-FLAG antibody **(Lines 717-718, 344-346)**. For bisulfite sequencing **(Figure 5)**, we have added detailed information on the positive and negative controls to the Figure legend **(Lines 727, 730-731)**. These additions clarify the negative controls employed and support the specificity of the observed binding and methylation changes.

***Comment 4**: While the methods section is generally detailed, please ensure that all primers (e.g., for qPCR and bisulfite sequencing) and PCR conditions are included in the manuscript or supplementary tables for full reproducibility.*

Response: Primers and PCR conditions are provided in Table S4 (for ChIP-qPCR) and Table S5 (for COBRA and bisulfite sequencing).

***Comment 5**: The conclusions are well supported by the data presented. The manuscript effectively shows that AID binds to the NINJ2 promoter/enhancer and reduces DNA methylation, which correlates with increased gene expression. A limitation acknowledged by the authors is that AID-KO mice exhibit systemic immune alterations that might indirectly affect epithelial regeneration. A discussion on how these systemic effects might influence the interpretation of the results would further strengthen the manuscript.*

Response: Thank you. We have discussed the potential influence of systemic immune alterations, such as reduced IgA production and dysbiosis, on epithelial regeneration in AID-deficient mice **(Page 13, Lines 306-309).**

"Although our *in vitro* experiments clearly demonstrate that AID binds to the *NINJ2* promoter and induces DNA demethylation, it is important to consider that the delayed regeneration observed in AID-deficient mice may also be influenced by systemic immune alterations such as reduced IgA production and dysbiosis."

***Comment 6**: To more fully establish the causality between NINJ2 expression and tissue*

© 2026. Published by The Company of Biologists under the terms of the Creative Commons Attribution License (https://creativecommons.org/licenses/by/4.0/).

regeneration, an experiment using NINJ2 knockdown in the AID-OP model (or in vivo) could be considered in future studies. However, I recognize that this may be beyond the current scope.
Response: We appreciate this insightful suggestion. We agree that this would be an important future direction, and we will consider conducting such experiments in subsequent studies.

Comment 7: The title and abstract accurately reflect the content of the manuscript and avoid hyperbole. Overall, the manuscript is clear and well written, though minor editorial polishing (e.g., consistent gene/protein nomenclature) would further improve readability.
Response: This is a very helpful comment. All relevant changes have been highlighted in the revised manuscript to facilitate review (see marked version).
(Lines revised: 31, 34, 37, 53-56, 81, 89-90, 102-103, 124, 147-152, 154, 156, 158-159, 161-163, 166-167, 169, 175, 179-181, 183, 185-186, 189, 197, 199, 201-202, 231, 241, 246, 264, 268, 284, 287, 289-294, 304, 343, 427-428, 449, 708-709, 712, 716-717, 722, 726-727, 731-732, 736, 749, 751, 753).

Third decision letter

MS ID#: bio.062001R2

MS Title: Activation-induced cytidine deaminase promotes DNA demethylation and expression of the Ninjurin-2 gene

Authors: Toshiyuki Okada, Yusuke Toyoda and Shigeaki Saitoh

I have now reached a decision on the above manuscript.

The reviewer reports are shown at the bottom of this email.

As you will see, the reviewers gave favourable reports, but raised some critical points that will require amendments to your manuscript. I hope that you will be able to carry these out, because we would like to be able to accept your paper.

At this stage, we also ask you to ensure your manuscript complies with our formatting guidelines - please see our manuscript preparation guidelines for details. Provided you are able to fully address the referees' comments, we are positive about publication of your paper (we accept over 95% of revision submissions) and therefore hope you won't mind any extra work involved in reformatting your manuscript at this point.

Please upload both a 'clean' version of your Word file, along with a highlighted version clearly showing where you have made changes in the revised manuscript. Please avoid using 'Track changes' in Word files as these are lost in PDF conversion.

I should be grateful if you would also provide a point-by-point response detailing how you have dealt with the points raised by the reviewers in the 'Response to Reviewers' box. Please attend to all of the reviewers' comments. If you do not agree with any of their criticisms or suggestions please explain clearly why this is so.

Reviewer 1

Comments for the author

Major Comments

Controls - Some key assays (ChIP-qPCR, COBRA/BSP) lack negative and input controls. These should be included or clarified.

© 2026. Published by The Company of Biologists under the terms of the Creative Commons Attribution License (https://creativecommons.org/licenses/by/4.0/).

Statistical analysis - Please specify whether multiple testing corrections were applied for microarray and qPCR data. Several comparisons use borderline p-values without adjustment.

Reproducibility - Biological replicates are limited (n = 3 in mouse studies; single CRC line used for mechanistic validation). Additional replicates or justification are needed.

Mechanistic link - The causal connection between AID-mediated demethylation and NINJ2-driven recovery is not fully established. A rescue experiment (e.g., NINJ2 re-expression in AID-KO mice) would strengthen the conclusions.

Alternative explanations - The discussion should address possible indirect regulation (e.g., NF-κB activation, inflammatory signaling) and consider potential oncogenic risks of AID overexpression in epithelial cells.

Minor Comments

Provide full experimental details: siRNA sequences, antibody catalog numbers, and exact n for animal studies.

Clarify figure legends to specify biological vs technical replicates and controls used.

Ensure consistent nomenclature (e.g., AID vs AICDA) throughout the text.

Improve clarity in the Results by summarizing key findings before detailed description of individual assays.

Reviewer's Responses to Questions

Experimental quality
Does each figure have the proper controls?
If 'No', please indicate reasons in Comments for Author box below.
Reviewer #1:

- No

Were the data analyzed using appropriate statistical tests?
If 'No', please indicate reasons in Comments for Author box below.
Reviewer #1:

- No

Reproducibility
Were experiments performed using adequate number of biological replicates?
If 'No', please indicate reasons in Comments for Author box below.
Reviewer #1:

- No

Does the methods section provide sufficient detail to permit reproducibility?
If 'No', please indicate reasons in Comments for Author box below.
Reviewer #1:

- No

Completeness
Are the manuscript's conclusions supported by the data?
If 'No', please indicate reasons in Comments for Author box below.
Reviewer #1:

- No

Scholarship
Do the authors cite and discuss the merits of data that would argue for and against their

© 2026. Published by The Company of Biologists under the terms of the Creative Commons Attribution License (https://creativecommons.org/licenses/by/4.0/).

conclusion?
If 'No', please indicate reasons in Comments for Author box below.
Reviewer #1:

- No

Does the manuscript title & abstract accurately reflect the contents of the manuscript, without hyperbole?
If 'No', please indicate reasons in Comments for Author box below.
Reviewer #1:

- Yes

Third revision

Author response to reviewers' comments

We sincerely thank the Editor and Reviewer #1 for their careful evaluation of our manuscript and for the constructive comments provided. These suggestions have substantially improved the clarity and scientific rigor of our manuscript. Below, we respond to each comment point by point. All revisions have been highlighted in the revised manuscript.

Response to Reviewer 1:

[Major Comments]

1. ***Controls - Some key assays (ChIP-qPCR, COBRA/BSP) lack negative and input controls. These should be included or clarified.***

Response: We addressed this concern by updating the figures and clarifying the analyses as follows.

- **ChIP-qPCR (Figure 4B):**
  We updated the data to include a ***No Antibody*** (No Ab) condition as a negative control for both Control#3 and AID-FLAG#1 cells. To rigorously compare the four experimental conditions, we performed a one-way ANOVA followed by Tukey's multiple-comparisons test. These updates are described in the revised legend of Fig. 4 (lines 750-757). The revised analysis demonstrates that enrichment of AID at the *NINJ2* promoter is statistically significant (P = 0.0011) and specific relative to controls, which is also stated in the revised Results text (lines 180-182).
- **Input normalization (Figure 4B)***:*
  As indicated in the axis label (Relative enrichment % Input), all ChIP-qPCR data were normalized to input DNA to account for variation in chromatin amounts.
- **COBRA/BSP (Figure 5):**
  To clarify the controls used in the methylation analyses, we revised the labels in Fig. 5. Fully methylated DNA is now labeled as *Positive Control* (formerly MD), and unmethylated DNA is labeled as *Negative Control* (formerly NMD). These controls are explained in the legend of Fig. 5A (lines 762-765).

2. ***Statistical analysis - Please specify whether multiple testing corrections were applied for microarray and qPCR data. Several comparisons use borderline p-values without adjustment.***
   Response: Thank you for pointing this out. We clarified the statistical approaches used in the revised manuscript.

- **Microarray analysis:**
  We did not apply global multiple-testing corrections (e.g., FDR). Instead, we employed a stringent Z-score cutoff (≥ 2.0) to identify genes with robust expression changes, which were subsequently validated by RT-qPCR. This approach is described in the Methods section (lines 385-395). In addition to the revisions made in response to your comments, we have provided the complete list of genes identified in our microarray analysis (*Z*-score ≥ 2.0 or ≤ -2.0) as a Supplementary Material (line 115).
- **RT-qPCR and ChIP-qPCR:**

© 2026. Published by The Company of Biologists under the terms of the Creative Commons Attribution License (https://creativecommons.org/licenses/by/4.0/).

For datasets involving comparisons among three or more experimental groups (e.g., Figure 2B and Figure 4B), we utilized one-way ANOVA followed by Tukey's multiple-comparisons test to account for multiple comparisons. Statistical significance is indicated in the corresponding figure legends (lines 729–737, lines 750–757). We have also updated the Statistics section in the Materials and Methods to explicitly state that one-way ANOVA followed by Tukey's test was used for multiple group comparisons (lines 525-527).

- **Animal Studies (Figure 6 and Figure S4):**
  We revised the manuscript to report exact *P*-values (e.g., $P = 0.049$) rather than approximate thresholds for *in vivo* experiments, providing more precise reporting for borderline results (line 217, lines 218-219, lines 221-222, lines 225-227).

3. ***Reproducibility - Biological replicates are limited (n = 3 in mouse studies; single CRC line used for mechanistic validation). Additional replicates or justification are needed.***

Response: We appreciate the reviewer's concern regarding reproducibility. We have clarified our experimental design and sample sizes in the revised manuscript.

- **Mouse studies:**
  We apologize for the confusion caused by the lack of clarity in our original description. The previously stated $n = 3$ referred to the number of independent experiments, not the total number of animals. We have revised the figure legends to clearly indicate that the data represent results pooled from at least three independent experiments (line 775-783). Accordingly, the total sample sizes for the key *in vivo* experiments are $n = 8$-12 per group (e.g., Fig. 6A: WT n=8, AID-KO n=12). We have also explicitly defined these selection criteria in the Materials and Methods section (lines 477-484), consistent with the description in the Results section, stating that mice exhibiting a 10–20% reduction in body weight were specifically selected to minimize inter-individual variability.
- **Single CRC cell line:**
  We specifically selected cell lines lacking detectable endogenous AID expression to establish a clean gain-of-function system. Although DLD-1 and T84 cells were initially considered, T84 cells were excluded because of extremely slow growth and low transfection efficiency. Therefore, DLD-1 was selected as the optimal model, and we have added this rationale to the Results section (lines 105–108). Importantly, the key findings obtained in DLD-1 cells were consistently supported *in vivo* using AID-deficient mice (Fig. 2 and Fig. 4).

4. ***Mechanistic link - The causal connection between AID-mediated demethylation and NINJ2-driven recovery is not fully established. A rescue experiment (e.g., NINJ2 re-expression in AID-KO mice) would strengthen the conclusions.***

Response: We thank the reviewer for this insightful suggestion. We agree that an *in vivo* rescue experiment would further strengthen the mechanistic link. While such experiments are beyond the scope of the present study, we acknowledge this limitation and expanded the Discussion to address it explicitly (lines 294-297).

5. ***Alternative explanations - The discussion should address possible indirect regulation (e.g., NF-κB activation, inflammatory signaling) and consider potential oncogenic risks of AID overexpression in epithelial cells.***

Response: Thank you for this valuable suggestion. We revised the Discussion to address both points.

- **Indirect Regulation:**
  We note that *NINJ2* expression may also be influenced by inflammatory signaling pathways, such as NF-κB activation, independently of AID (lines 297–298).
- **Oncogenic Risks:**
  We expanded the Discussion to describe potential oncogenic risks associated with ectopic AID expression, including evidence that aberrant AID expression in IBD patients can promote somatic mutations in the *TP53* tumor suppressor gene and contribute to malignant transformation (lines 299–306).

[Minor Comments]

1. ***Provide full experimental details: siRNA sequences, antibody catalog numbers, and exact n for animal studies.***

Response: We have included all the requested details in the Materials and Methods section:

- **siRNA Sequence:** (lines 367–369)

© 2026. Published by The Company of Biologists under the terms of the Creative Commons Attribution License (https://creativecommons.org/licenses/by/4.0/).

The sequence of the human *AICDA*-targeting siRNA was added (5′-GGACUUUGAUAGCAACUUC-3′; Dharmacon, siGENOME, Cat. #021409-02, duplex #2). Since the sequences of the control siRNAs are proprietary, we provided the product names and catalog numbers.

- **Antibodies:** (lines 508–513)
  Catalog numbers and dilution ratios for all antibodies were rechecked and clarified as needed.
- **Exact sample numbers for *animal studies*:** (lines 733-734, line 775, line 778)
  As noted above, exact *n* values (e.g., WT *n*=5, AID-KO *n*=9) are now provided in the figure legends.

2. ***Clarify figure legends to specify biological vs technical replicates and controls used.***

Response: Figure legends and **Supplementary Information** were revised to clearly distinguish biological replicates (pooled from independent experiments) from technical replicates (lines 725-726, lines 735-737, lines 746-747, lines 753-755, and 782-783). Technical replicates are specified where applicable (e.g., Fig. 2 and Fig. 3; line 734 and line 744). **For Figure 1C, S2A, and S2B**, we revised the figure legends to specify the exact sample size for each gene rather than providing a range (e.g., *n* = 4 for *NINJ2* and *HIST1H1E*, *n* = 3 for *FOXP1*) (lines 724-725). We also explicitly described the specific controls used in each experiment, such as *No ab* (no antibody) for ChIP (Figure 4) and "Positive/Negative control" for methylation assays (Figure 5).

3. ***Summarizing key findings (Clarity of Results). Ensure consistent nomenclature (e.g., AID vs AICDA) throughout the text. Improve clarity in the Results by summarizing key findings before detailed description of individual assays.***

Response: Thank you for your valuable suggestions.
We standardized the nomenclature to AID (italicized for the gene, non-italicized for the protein) throughout the manuscript and improved the clarity of the Results section by adding summary sentences at the beginning of each subsection to highlight key findings before detailed descriptions (lines112-114, lines 136-141, lines 153-155, line 168, lines 170-171, lines 187-188, lines 190-191, line 211-213, line 164-165, line 203-204). Paragraph breaks were also added to improve logical flow (lines 110 and 122, line 413, line 423, line 429, line 434).

Fourth decision letter

MS ID#: bio.062001R3

MS Title: Activation-induced cytidine deaminase promotes DNA demethylation and expression of the Ninjurin-2 gene

Authors: Toshiyuki Okada, Yusuke Toyoda and Shigeaki Saitoh

I have now reached a decision on the above manuscript.

The reviewer reports are shown at the bottom of this email.

As you will see, the reviewers gave favourable reports, but raised some critical points that will require amendments to your manuscript. I hope that you will be able to carry these out, because we would like to be able to accept your paper.

At this stage, we also ask you to ensure your manuscript complies with our formatting guidelines - please see our manuscript preparation guidelines for details. Provided you are able to fully address the referees' comments, we are positive about publication of your paper (we accept over 95% of revision submissions) and therefore hope you won't mind any extra work involved in reformatting your manuscript at this point.
Please upload both a 'clean' version of your Word file, along with a highlighted version clearly showing where you have made changes in the revised manuscript. Please avoid using 'Track changes' in Word files as these are lost in PDF conversion.

© 2026. Published by The Company of Biologists under the terms of the Creative Commons Attribution License (https://creativecommons.org/licenses/by/4.0/).

I should be grateful if you would also provide a point-by-point response detailing how you have dealt with the points raised by the reviewers in the 'Response to Reviewers' box. Please attend to all of the reviewers' comments. If you do not agree with any of their criticisms or suggestions please explain clearly why this is so.

Thank you for the carefully revised manuscript. The study provides a coherent set of observations linking inflammation-associated AID expression in colonic epithelial contexts to (i) increased NINJ2 transcription, (ii) reduced methylation at the NINJ2 promoter/enhancer region, and (iii) delayed recovery from DSS-induced injury in AID-deficient mice. The experimental workflow is generally well described and the core conclusions are largely supported. I have a few remaining points that would further strengthen rigor and interpretability:

Major points (rigor/clarity)

Microarray/statistics framing (screening vs inference).
The transcriptome screen uses a Z-score threshold to define AID-responsive genes. This approach can be fine as a discovery filter, but please make the statistical framing explicit:

Clarify that the microarray is hypothesis-generating and that claims of differential expression are primarily supported by independent RT-qPCR validation of representative targets.

If feasible, briefly note whether replicate arrays exist; if not, state this limitation transparently and point readers to the GEO deposit as supporting transparency.

Replicate definitions and n reporting (biological vs technical).

Please ensure each figure legend unambiguously reports:

Biological n (independent cultures; number of animals per group), and

Technical replicates (qPCR duplicates/triplicates; gel repeats; subclones in BSP).
This is particularly important where BSP subclones are used (subclones increase sequencing depth but do not substitute for independent biological replicates).

ChIP-qPCR controls and normalization shown explicitly.
Because ChIP is central to the "direct binding" claim, please keep the control structure clearly visible in the final figure/legend:

Input normalization strategy,

A negative immunoprecipitation control (e.g., mock/No-antibody or IgG where applicable), and

Clear indication that enrichment is specific to AID-FLAG versus vector control.

Causal boundary: NINJ2 as mediator of recovery.
The in vivo phenotype (delayed recovery in AID-KO) is compelling, and the AID→NINJ2 epigenetic regulation is supported. However, a direct demonstration that NINJ2 mediates recovery (e.g., rescue or re-expression) is not shown. Please keep this as a clearly stated limitation (and avoid wording that implies definitive causality beyond the presented data).

Minor points (presentation)

Please ensure consistent gene/protein nomenclature throughout (e.g., italicized gene symbols where appropriate; human vs mouse symbols).

Where P values are near threshold, consider reporting exact P values and the test used in the legend for transparency.

If not already present, a brief statement on randomization/blinding (or why not feasible) for mouse scoring would be helpful.

© 2026. Published by The Company of Biologists under the terms of the Creative Commons Attribution License (https://creativecommons.org/licenses/by/4.0/).

Overall, the revision is close to being fully locked from a rigor and clarity perspective; addressing the points above mainly requires explicit reporting and careful phrasing, rather than new experiments.

Reviewer's Responses to Questions

Experimental quality
Does each figure have the proper controls?
If 'No', please indicate reasons in Comments for Author box below.
Reviewer #1:

- Yes

Were the data analyzed using appropriate statistical tests?
If 'No', please indicate reasons in Comments for Author box below.
Reviewer #1:

- Yes

Reproducibility
Were experiments performed using adequate number of biological replicates?
If 'No', please indicate reasons in Comments for Author box below.
Reviewer #1:

- Yes

Does the methods section provide sufficient detail to permit reproducibility?
If 'No', please indicate reasons in Comments for Author box below.
Reviewer #1:

- Yes

Completeness
Are the manuscript's conclusions supported by the data?
If 'No', please indicate reasons in Comments for Author box below.
Reviewer #1:

- Yes

Scholarship
Do the authors cite and discuss the merits of data that would argue for and against their conclusion?
If 'No', please indicate reasons in Comments for Author box below.
Reviewer #1:

- Yes

Does the manuscript title & abstract accurately reflect the contents of the manuscript, without hyperbole?
If 'No', please indicate reasons in Comments for Author box below.
Reviewer #1:

- Yes

Fourth revision

© 2026. Published by The Company of Biologists under the terms of the Creative Commons Attribution License (https://creativecommons.org/licenses/by/4.0/).

Author response to reviewers' comments

We sincerely thank the Editor and Reviewer #1 for their careful evaluation of our manuscript and for the constructive comments provided. These suggestions have substantially improved the clarity and scientific rigor of our manuscript. Below, we respond to each comment point by point. All revisions have been highlighted in the revised manuscript.

Response to Reviewer 1:

[Major Comments]

1. Microarray/statistics framing (screening vs inference).
The transcriptome screen uses a Z-score threshold to define AID-responsive genes. This approach can be fine as a discovery filter, but please make the statistical framing explicit: Clarify that the microarray is hypothesis-generating and that claims of differential expression are primarily supported by independent RT-qPCR validation of representative targets. If feasible, briefly note whether replicate arrays exist; if not, state this limitation transparently and point readers to the GEO deposit as supporting transparency. Replicate definitions and n reporting (biological vs technical).
Response: The text has been revised to explicitly state that the microarray analysis was conducted to identify candidate genes whose expression levels were affected by AID overexpression. The expression levels of these candidates were subsequently examined by RT-qPCR **(lines 111-113)**. To ensure transparency, we added a statement that the microarray analysis was conducted once without biological replicates. The data are deposited in GEO with the accession number GSE271083, which is indicated in the **legend of Figure 1** and the **Data Availability section** **(lines 733-734 and 560-562)**.

2. Please ensure each figure legend unambiguously reports:
Biological n (independent cultures; number of animals per group), and Technical replicates (qPCR duplicates/triplicates; gel repeats; subclones in BSP). This is particularly important where BSP subclones are used (subclones increase sequencing depth but do not substitute for independent biological replicates).
Response: As suggested, the figure legends have been revised, and the number of the biological replicates (*n*, independent cultures or individual mice) and technical replicates (qPCR wells or sequencing clones) are specified. The specific revisions to the manuscript are as follows:

- qPCR and ChIP-qPCR (**Figures 1-4**): We clearly defined independent cultures as biological replicates and specified the technical replicates (single, duplicate, or triplicate wells) for each assay **(lines 739-743, 749-751, 762-764, 774-775, and 777-779).**
- Bisulfite sequencing **(Figure 5)**: As stated in the revised figure legend **(lines 789-791 and 794-796)**, the genomic DNA used for the analysis was isolated from representative single cultures of the cell lines. Eight subcloned DNA fragments were sequenced in each group. For controls, five subcloned DNA fragments were sequenced.
- Mouse Studies **(Figure 6)**: The number of mice used in the experiments is presented in the figure. The revised figure legend states, "*n* indicates the number of mice used for each genetic background" **(line 804).**

3. ChIP-qPCR controls and normalization shown explicitly.
Because ChIP is central to the "direct binding" claim, please keep the control structure clearly visible in the final figure/legend: Input normalization strategy, A negative immunoprecipitation control (e.g., mock/No-antibody or IgG where applicable), and Clear indication that enrichment is specific to AID-FLAG versus vector control.
Response: As suggested, we revised the Materials and Methods section, as well as the legend for **Figure 4** to explicitly describe the experimental design and data processing. Specifically, we clarified the following points:

- **Input Normalization:** The abundance of *NINJ2* in immunoprecipitated chromatin DNA was normalized by dividing it by the abundance in the input DNA, and the results are presented as "% Input." To provide further clarity on our normalization strategy, we have explicitly added the exact formula used for the calculation to the Materials and Methods section: % Input = 100 × 2^[Ct(Input) - log2(Dilution factor) - Ct(ChIP)]. This point is now clarified in

© 2026. Published by The Company of Biologists under the terms of the Creative Commons Attribution License (https://creativecommons.org/licenses/by/4.0/).

lines 447-449 and 775-777.

- **Negative Controls:** Two types of negative controls are included in the ChIP experiment: mock immunoprecipitation without an antibody (no Ab control) and cells that do not express AID-FLAG (control#3). As stated in the revised text (lines 772-774), the no ab control clearly shows that *NINJ2* DNA does not bind to the magnetic beads. A statistical comparison of the amounts of immunoprecipitated *NINJ2* from AID-FLAG#1 and control#3 confirms that *NINJ2* DNA specifically co-precipitates with FLAG-tagged AID.

4. Causal boundary: NINJ2 as mediator of recovery.
The in vivo phenotype (delayed recovery in AID-KO) is compelling, and the AID→NINJ2 epigenetic regulation is supported. However, a direct demonstration that NINJ2 mediates recovery (e.g., rescue or re-expression) is not shown. Please keep this as a clearly stated limitation (and avoid wording that implies definitive causality beyond the presented data).
Response: We appreciate the reviewer's assessment that the *in vivo* phenotype is compelling. While the delayed recovery in AID-deficient mice and the epigenetic regulation of *NINJ2* by AID suggest that *NINJ2* mediates epithelial recovery, as the reviewer pointed out, our study does not provide direct evidence of a causal relationship between them. Accordingly, we revised the text to clarify this limitation and avoid wording that implies definitive causality. We also discuss possible mechanisms connecting AID, NINJ2, and tissue recovery (lines 230-232, 235-238, 268-270, 279-282, 288-290, 292-295, 299-303, and 327-333).

[Minor Comments]
1. Please ensure consistent gene/protein nomenclature throughout (e.g., italicized gene symbols where appropriate; human vs mouse symbols).
Response: We have carefully reviewed the entire manuscript and ensured that gene and protein nomenclature is consistent throughout. Specifically, we have verified that all gene symbols (e.g., *NINJ2*, *AID*, *TP53*) are appropriately italicized, and that human and mouse symbols are correctly distinguished according to standard nomenclature guidelines (lines 42, 54, 60, 105, 246, 268, and Supplemental Information). We have also corrected typographical and spelling errors, and standardized the terms "colon cancer" to "colorectal cancer (CRC)" and "dextran sodium sulfate" to "dextran sulfate sodium (DSS)" for consistency (lines 100, 102, 129, 147, 167, 337, 353, 407, 423, 438, 443, 446, 479, 499, 499-500, 731, and 784).

2. Where P values are near threshold, consider reporting exact P values and the test used in the legend for transparency.
Response: To improve data transparency as suggested, we have updated the text and figure legends to report the exact *P* values, particularly those near the 0.05 threshold, such as $P = 0.049$ in Fig. 2B (line 753), $P = 0.057$ and $P = 0.047$ in Fig. 3 (lines 759 and 765-766), and $P = 0.049$ in Fig. 6B (line 807). We have also clearly stated the statistical tests used for these analyses (e.g., ordinary one-way ANOVA with Tukey's multiple comparison test, nonparametric Mann-Whitney U test, or Pearson correlation analysis) in the respective figure legends.

3. If not already present, a brief statement on randomization/blinding (or why not feasible) for mouse scoring would be helpful.
Response: We have added a brief statement to the Materials and Methods section (under "Mice and treatment with dextran sodium sulfate") to clarify that the daily monitoring and scoring of the mice, including the calculation of the disease activity index (DAI), were performed blindly (lines 499-500).

Fifth decision letter

MS ID#: bio.062001R4

MS Title: Activation-induced cytidine deaminase promotes DNA demethylation and expression of the Ninjurin-2 gene

© 2026. Published by The Company of Biologists under the terms of the Creative Commons Attribution License (https://creativecommons.org/licenses/by/4.0/).

Authors: Toshiyuki Okada, Yusuke Toyoda and Shigeaki Saitoh

I am happy to tell you that your manuscript has been accepted for publication in Biology Open, pending our standard publication integrity checks. It was accepted on 12th May 2026.

© 2026. Published by The Company of Biologists under the terms of the Creative Commons Attribution License (https://creativecommons.org/licenses/by/4.0/).
